# Dietary patterns and socioeconomic disadvantage: an analysis of food consumption patterns and their determinants in Cochabamba, Bolivia

**DOI:** 10.1186/s44263-025-00221-2

**Published:** 2025-11-20

**Authors:** Rodrigo Álvaro Quispe Condori, María del Carmen Ledo García, Rodrigo Karlop Arce Cardozo

**Affiliations:** 1 School of Global Studies, Gothenburg University, Gothenburg, Sweden; 2 Planning and Management Center, San Simon University, Cochabamba, Bolivia; 3 Department of Epidemiology and Global Health, Umeå University, Umeå, Sweden; 4 Biomedical and Social Research Institute, San Simon University, Cochabamba, Bolivia

**Keywords:** Dietary pattern, Multivariate analysis, Socioeconomic disadvantage, Bolivia

## Abstract

**Background:**

In low- and middle-income countries, socioeconomic disadvantages often shape the dietary patterns of the people living in them, frequently towards a diet rich in calories and poor in nutrients. In Bolivia, very little is known about the dietary patterns of household; in this regard, this study aimed to identify the dietary patterns of the population in the Municipality of Cochabamba and to analyze their relationship with socioeconomic, and demographic characteristics.

**Methods:**

We conducted a cross-sectional observational study, with data collection conducted between October 2022 and March 2023. The sampling was done through a representative complex survey design of the Cochabamba urban population (*n* = 4496). Dietary patterns were constructed by combining principal component analysis and cluster analysis. Given their multidimensionality, socioeconomic, and demographic variables, the prevalence of cardio-metabolic diseases and food preferences were synthesized in a standardized index. This index was constructed by applying principal component analysis and multiple correspondence analysis. The association between dietary patterns and their determinants was analyzed using multivariate logistic regression considering a significance level of 0.05.

**Results:**

Two distinct dietary patterns were identified: whole-grain and plant-based foods (37%) and a calorie-rich but nutrient-poor diet (63%). This last one was statistically significantly associated with belonging to a medium–low socioeconomic stratum (*p* < 0.01), poor housing conditions (*p* < 0.05), informal occupational category (*p* < 0.05), food preferences for processed foods (*p* < 0.01), and a marginal association of interest with the prevalence of cardiometabolic diseases (*p* < 0.1). In this study, the peripheral areas of Cochabamba concentrated the population with the highest adherence to the calorie-rich but nutrient-poor diet.

**Conclusions:**

To our knowledge, this is the first study to analyze the relationship between socioeconomic disadvantages and dietary patterns in Cochabamba, Bolivia. Belonging to a lower socioeconomic group was the main determinant of dietary patterns. The presence of a dietary pattern composed mainly of highly processed foods highlights the need to implement public policy measures and interventions to limit the supply of these products, given their expected negative impact on health.

**Supplementary Information:**

The online version contains supplementary material available at 10.1186/s44263-025-00221-2.

## Background

One of the problems facing the nations of the world is the increasing trend in the consumption of fast food and ultra-processed foods [1–6]. This has been shown to be deteriorating people’s health and increasing the prevalence of non-communicable diseases (NCDs). NCDs together account for 74% of deaths worldwide and constitute a significant burden on health systems, especially in low- and middle-income countries (LMICs), which account for three-quarters of NCD deaths [7, 8]. Some researchers have described this phenomenon as “fast food genocide” [9] or as part of a “nutritional transition” [10] and are related to dietary and nutritional changes driven by economic, social, and demographic changes.

In the global south, many countries [11–14], among them Bolivia [15], are going through a transition marked by an accelerated process of urbanization, greater economic growth, and changes in lifestyles motivated by globalization [11, 16, 17], whose effects are accentuated in medium–low socioeconomic communities [18].

In the case of the Municipality of Cochabamba—which encompasses the city of Cochabamba in central Bolivia—the extension of the urban sprawl has led to a fragmented city configuration with marked manifestations of socio-spatial segregation and physical marginality [19]. The northern and central areas of the city concentrate the population belonging to the medium–high stratum, who have full access to basic services, while the peripheral areas, particularly the southern periphery, bring together medium–low socioeconomic groups [20]. The pattern of segregation observed in Cochabamba not only reflects socioeconomic differences but also shifts in the population’s dietary patterns (DPs), which have evolved towards a low-cost diet, predominantly composed of pre-cooked and processed foods, to the detriment of traditional and homemade meals, whose popularity has progressively decreased over time [21–23].

In accordance with the above, DPs are influenced by socioeconomic, demographic, and spatial factors [24–26], which makes their characterization important. This study attempts to fill a gap in the literature on the relationship between socioeconomic context and DPs in urban populations in Bolivia. Likewise, the results aim to serve as a valuable resource for understanding the connections between DPs and health, as well as for informing public policy decisions and interventions aimed at reducing food inequalities and promoting healthy eating behaviors in this population.

## Methods

### Study setting

Bolivia is a landlocked country located in the center of South America, with an estimated population of 12.33 million in 2024 [27]. It falls within the group of low- and middle-income countries and ranks 120 out of 193 on the Human Development Index [28]. Cochabamba is one of the nine departments of Bolivia, and the municipality of the same name concentrates 40% of the urban population; it is one of the leading centers for the trade of food in the region since it borders the most populated municipalities, including Colcapirhua, Sacaba, Tiquipaya, Quillacollo, Arbieto, and Santivañez [29].

**Fig. 1:**
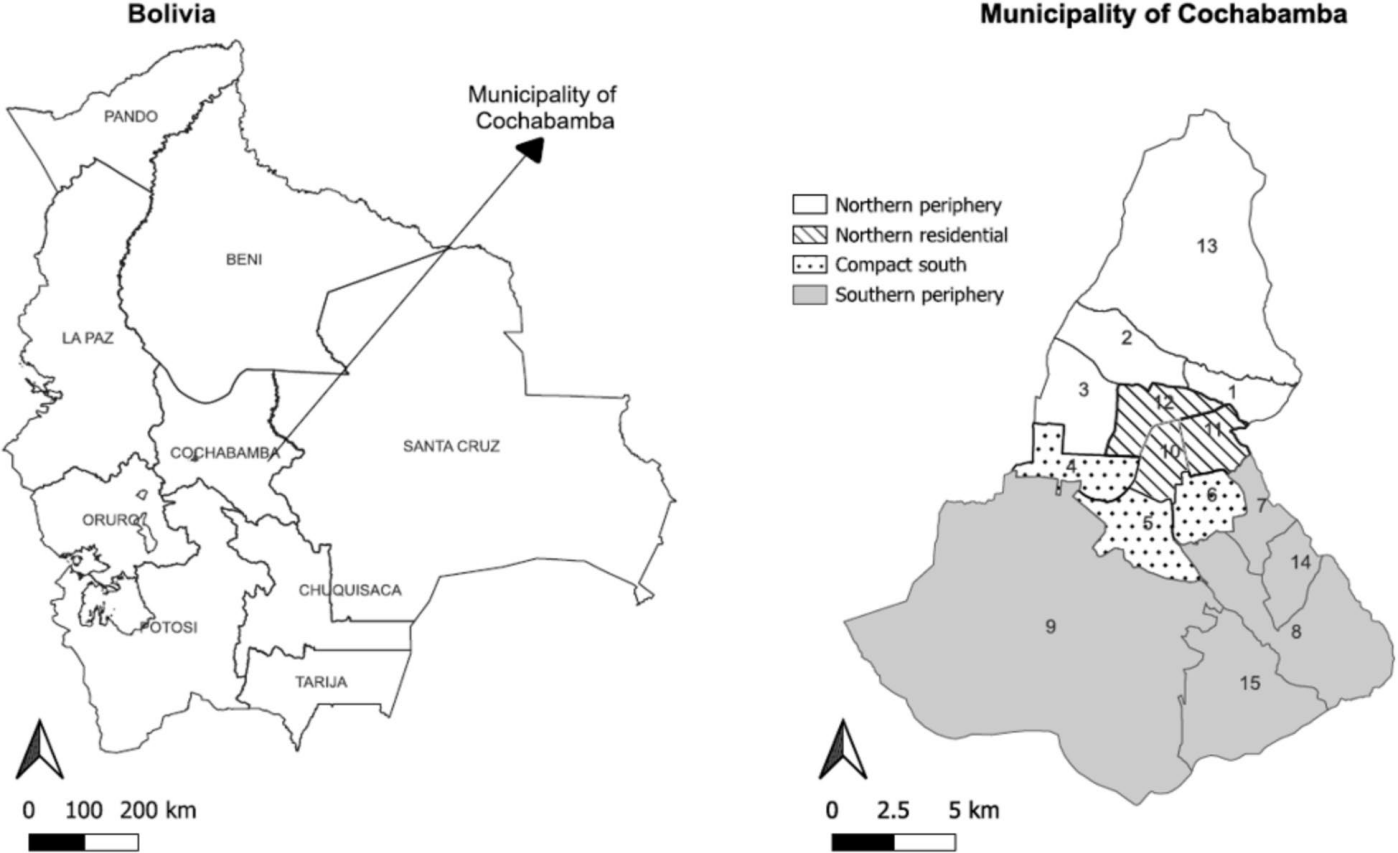
Location of the municipality of Cochabamba in the Bolivia (left) and districts of the municipality (right), 2024. *Map developed in Quantum Geographic Information System (QGIS) using open-access data from the Municipality of Cochabamba*

The municipality of Cochabamba has an area of 1150 km^2^ (Fig. 1). Politically and administratively, it is divided into 15 urban districts. The spatial distribution of these districts reveals a process of social exclusion and vulnerability [19], driven by internal migration flows [30] that have fragmented the city [31]. These disparities allowed for the grouping of districts into four zones: districts 1, 2, 3, 13, and 7, 8, 9, 14, and 15 form the northern and southern peripheral zones, while districts 4, 5, 6, and 10, 11, 12 correspond to the “compact south” and “northern residential” zones, respectively. The latter two zones, unlike the others, are characterized by complete access to basic services and house populations of executives, professionals, and non-manual employees [19].

### Study design

This study utilized data from the Nutrition and Development Survey (NDS), a complex survey developed based on the Urbanization, Employment, Migration, and Quality of Life survey [32] and the Access to Drinking Water Services survey [33], conducted by the Planning and Management Center (CEPLAG) at the San Simon University (UMSS). The NDS survey was implemented between October 2022 and March 2023.

**Fig. 2:**
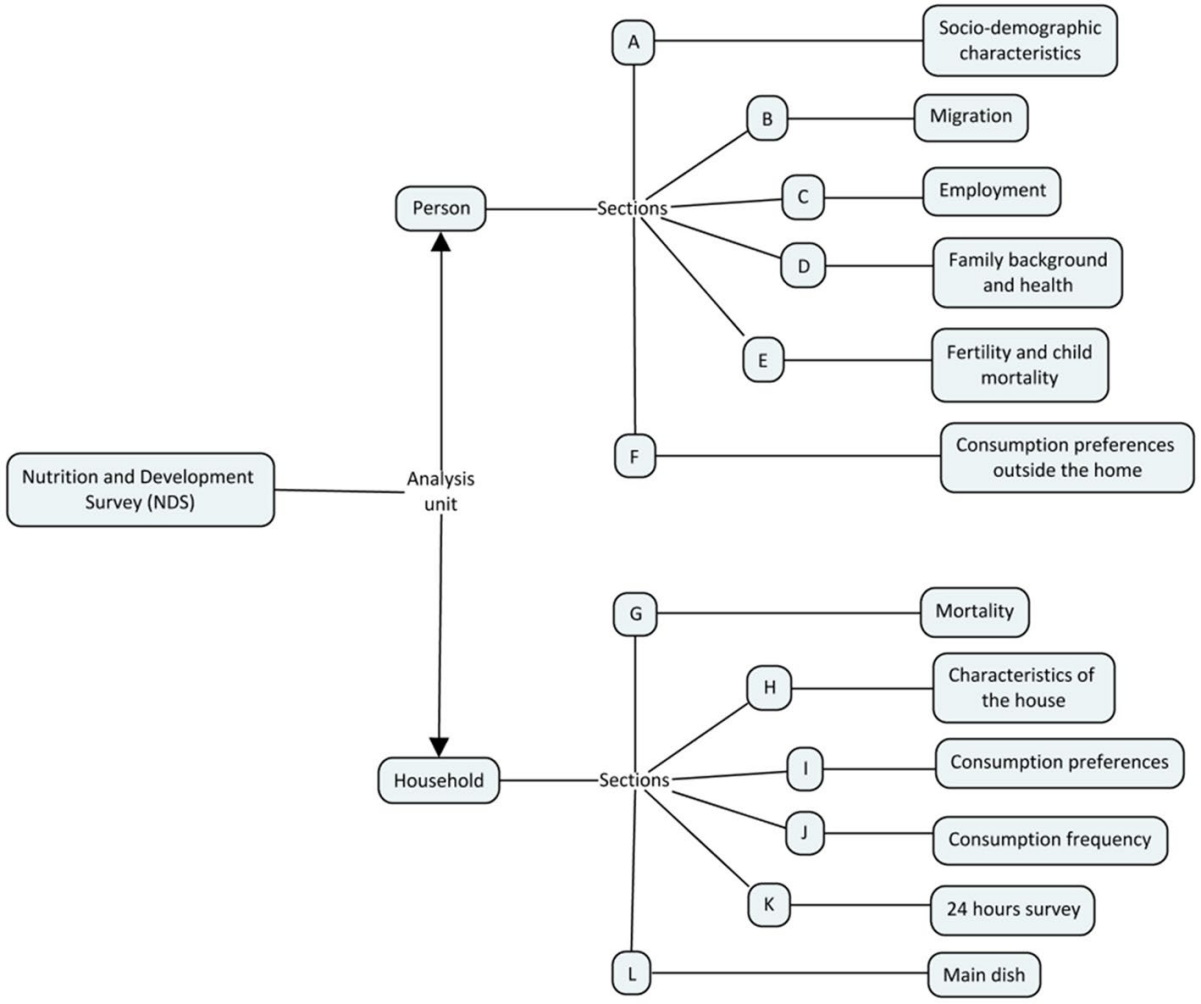
Structure of the Nutrition and Development Survey (2023), households in Cochabamba, Bolivia

This survey is organized into 12 sections. The first six sections retrieve information on each household member through a key informant, while the remaining sections focus on collecting household data on aspects of living standards, employment, household demographics, housing conditions, food consumption, and other relevant factors (see Fig. 2).

The primary sampling unit (PSU) was defined at the household level, and since updated data are not available, the housing population was estimated based on the population of blocks by district for 2016/2017, using the 2012 National Population and Housing Census. This projection was developed considering that the ratio of homes per block between 2012 and 2022 in the areas under study did not change; this assumption is based on the estimation of the Shannon entropy metric [34].

Subsequently, households were selected using stratified probability sampling with optimal allocation, according to Neyman’s method, which assigns a greater number of observations to strata with greater variability in the variable of interest—in this case, access to drinking water—and fewer observations to those with less variability [35]. Thus, the sample size per district, in total, amounts to 4496 households and about 15,260 inhabitants as a representative sample of the target population.

### Dietary assessment

Dietary data were obtained by applying a qualitative Food Frequency Questionnaire (FFQ) validated for Bolivian adolescents [36]. Foods that are not typical of the valleys area were omitted and others that are frequently consumed in the study area were added according to the Household Survey developed by the National Institute of Statistics (INE, Instituto Nacional de Estadistica, in Spanish) [37]. The FFQ included 102 foods (see Supplementary material 1: Table S1 and Supplementary material 2), classified into four groups according to the NOVA classification [38]. This framework was used solely to describe, in qualitative terms, the types of foods that make up the identified DPs and to facilitate the interpretation and comparison of our findings with regional and global studies [5, 39–44] that link the consumption of processed and ultra-processed foods with chronic noncommunicable diseases [45]. Furthermore, this classification, being intuitive, facilitates the communication of results to those responsible for public policies [46].

To validate the FFQ, a 24-h recall was applied (as a reference method) on a subsample of 200 heads of households. This 24-h recall (R24) was collected by trained nutritionists during the months of September and October of 2023. Next, the amounts of food, energy, macronutrients, and micronutrients ingested, both for the FFQ and the R24, were calculated using the Bolivian Food Table Program [47]. The FFQ was validated by estimating and analyzing Pearson’s correlation coefficients, the intraclass correlation coefficient (ICC) adjusted for energy according to the Willet residual method [48], and Lin’s concordance correlation coefficient (CCC) [49]. Our results showed that the FFQ performed adequately for measuring energy, macronutrients (protein, fat, and carbohydrates), and some vitamins and minerals (B12, niacin, and iron). However, it was not accurate for micronutrients such as potassium, folate, and vitamin A (see Supplementary material 1: Table S2).

The identification of DPs was performed using a completely data-driven approach, based exclusively on reported food consumption, without imposing predefined categories.

### Explanatory variables

The measurements of the analysis categories of this study involve the combination of multiple variables; therefore, it was necessary to construct synthetic measures to capture the key dimensions of analysis. Principal component analysis (PCA) [50], multiple correspondence analysis (MCA) [51], and hierarchical cluster analysis (HCA) [52] were applied for this purpose. To facilitate interpretation, all indices were normalized to a range of 0 to 1. Table 1 presents the complete list of indices and indicators.

**Table 1:** Variables and analysis techniques used for the construction of indexes and indicators, based on Nutrition and Development Survey (2023) in Cochabamba, Bolivia

Indexes/indicators	Variables	Analysis techniques
Dietary pattern	Frequency of consumption of minimally processed, culinary processed, processed, and ultra-processed foods	PCA and HCA
Zone of residence	District of residence (recoded)	-
Biological sex	Biological sex of the head of household	-
Age	Age of head of household	-
Household size	Number of household members	-
Food expenditure	Expenditure on food within the household	-
	Expenditure on food outside the household	
Literacy status of household members	Age of household members	-
	Can read and write	
Diets	Number of diets followed	-
Socioeconomic strata	Household per capita income	HCA
	Years of education of the head of household	
	Employment status	
Socio-occupational stratum	Main occupation	MCA
	Current occupational category	
Migratory status index	Place of birth	MCA
	Place of residence 5 years ago	
	Zone of residence	
	Current place of residence	
Unmet basic needs index	Electricity	MCA
	Cooking room	
	Drinking water piped into the house	
	Toilets for private use	
	Public sewer inside the house	
Housing construction quality index	Wall material	MCA
	Roof material	
	Floor material	
Food culture index	Meals prepared per day	PCA
	Main meal of the day	
Food shopping space index	Place where you buy your food	PCA
	Types of food purchased at the popular market	
	Types of food purchased at the supermarket	
Food preference index	Types of foods most consumed	MCA
	Food attributes	
Index of diseases	Frequent health problems	PCA
	Disease diagnoses	
	Disease diagnoses (parents)	

*PCA* principal component analysis, *HCA* hierarchical cluster analysis, *MCA* multiple correspondence analysis

The target municipality was divided into four zones: “Northern residential,” “Northern periphery,” “Compact south,” and “Southern periphery,” based on the classification proposed by Ledo [19]. The age of the heads of household was classified in three categories (17–44, 45–65, and 65 and over). The biological sex of the head of household was categorized into two categories (male and female); household size was categorized into three groups (1–2 members, 3–4 members, and 5 or more members); food expenditures within and outside the household were summed to obtain a total expenditure and grouped into three categories (< $US 258, between $US 259 and 402 and > $US 402). The proportion of people over 18 years of age who read and write was categorized into two groups (illiterate and literate). The average diet counts of the types of diets that household members have tried were grouped into two categories (single diet and more than one diet).

The socioeconomic stratum was constructed using HCA, which considered three variables related to the head of household: income was classified into three groups according to the World Bank guide [53], educational level (years of formal education) and occupational status, classified into three groups: employed, unemployed, and retired. Once the HCA was applied, three strata were constructed: “low stratum,” “medium–low stratum,” and “medium–high stratum.”

The socio-occupational stratum was estimated from the MCA and considered the main occupation and the current occupation categories, according to the Bolivian Classification of Occupations [54]. A higher index score is associated with a predominance of manual activities, while lower scores indicate non-manual activities. The migration status index was constructed considering the household members’ place of birth, place of residence in the last 5 years, and current area of residence. After applying the MCA, a factor was used to identify that differentiates migrant households from non-migrant households. Thus, households with a higher score reported more migrant members. This index captures the migratory dynamics of direct internal migrants, i.e., households with heads of household born within other Bolivian regions who migrated from the place of their birth to the Municipality of Cochabamba [55]. The measure underscore Cochabamba’s prominence as a key destination for large-scale migration flows from urban, semi-urban, and rural areas of the Andean highlands [56, 57].

To characterize households according to the type of housing, two indices have been constructed using the MCA: the index of unmet basic needs composed of variables of access to basic services (ownership of a kitchen, electricity, drinking water, sewage, and garbage collection services) and the index of construction quality of the housing, which considers variables related to the quality of construction materials (roof, floor, and walls). In both cases, a dimension was identified that contrasts those homes that have full access to basic services and are built with high-quality materials (tile roofs, wooden floors, and clad walls) with homes that do not have all the basic services and are built with low-quality materials (coral roofs, cement or dirt floors, and unclad walls). Values close to 1 correspond to homes with full access to basic services and construction with high-quality materials, while values close to 0 reflect a lack of some services and the use of substandard materials.

Additionally, the food culture index was estimated, which is composed of variables related to the number of meals prepared on a regular day and the meal considered the main meal. A factor has been found that contrasts households in which food is prepared three times a day and breakfast is considered the main meal, versus households in which dinner is considered the main meal and food is prepared less than three times a day. Values close to 1 refer to a household that prepares three meals a day and considers breakfast as the main meal.

The Food Shopping Space Index was constructed to capture the relationship between the diversity of places where people purchase their food and the types of food they purchase. The construction of this index was possible by combining the following variables: number of places where people buy their food, types of food purchased in popular markets, and types of food purchased in supermarkets and the application of the CPA which, through a principal component (48% of the explained variance), revealed an opposition between those households that purchased their food in a few places, compared to others that purchased it in multiple supply spaces (popular markets, supermarkets, neighborhood stores, micro markets, etc.). Households reporting a value close to 1 purchase their food from a few places, while those with a value approaching zero purchase their food from multiple supply centers. The household food preference index was also calculated based on food attributes (taste, appearance, and weight control) and the most consumed food types, grouped according to the NOVA classification. In this case, a factor was identified that contrast households that prefer minimally processed foods, allowing them to control their body weight (values converging to 0), with households that prefer processed foods with pleasant taste and appearance (values close to 1).

Finally, an index has been constructed for the case of diseases where the variables of quantity of frequent diseases, quantity of chronic diseases, and quantity of parental diseases have been incorporated. The highest values refer to households with members suffering from chronic diseases (such as diabetes, hypertension, obesity, and kidney damage), as opposed to those that only suffer from frequent or common acute diseases (diarrheal or respiratory diseases).

### Statistical analysis

The data collected through the NDS were entered into SPSS v.29 for cleaning and construction of all the indexes and indicators presented in Table 1. Given the nature of the variables analyzed, these were constructed using three multivariate analysis techniques: MCA, PCA, and HCA. The first allows us to explore associations between multiple categorical variables and represent them as points in a low-dimensional Euclidean space [58]. The advantage of this method is that it does not require compliance with any assumptions about probability distributions, nor does it require establishing predefined relationships between variables [59]. PCA, on the other hand, allows a set of continuous variables, generally correlated, to be transformed into a small subset of new uncorrelated variables called “principal components,” whose importance is established as a function of the total variance explained [60]. HCA is a clustering method commonly used to classify large data sets into clusters of elements that are similar to each other and different from elements belonging to other clusters [61].

Dietary patterns, consistent with previous studies [62, 63], were identified using a two-step procedure. Initially, the PCA with varimax rotation was applied to the frequency of food consumption, organized in food groups according to their degree of processing [64], after verifying its applicability based on the evaluation of the Kaiser–Meyer–Olkin (KMO) measure—where values close to 1 indicate adequate correlations among variables and confirm the suitability of applying PCA—and the Barlett sphericity index (*P* < 0.05) [65]. Once the PCA was applied, factor scores were obtained for each household member based on the extracted components from the aggregated food groups. It was necessary to normalize each of these main components in a range from 0 to 1 using the min–max transformation. This normalization is needed since there is no upper limit for the consumption of each food group. Considering that the sample contains the maximum cases that could occur in the population, it is normalized so that 1 means the maximum consumption of a given food. The above guarantees that the primary source of variation is the frequency of consumption, which can differentiate the type of consumption of strata and hide DPs.

Once the indices were obtained, the K-means algorithm was applied to determine the optimal number of groups and the sum of squares criterion within the groups. Then, the same function was used to perform an HCA using Ward’s method; according to this method and using the elbow criterion on the sedimentation graph and the graphical analysis on the dendrogram [66], the ideal number of mutually exclusive groups is two DPs. The number of groups was verified to be adequate by comparing the quality of grouping between the two methods, using a multivariate analysis of variance (MANOVA) for each grouping, which yielded the lowest sum of squares for two groups. Based on these results, it is concluded that the number of clusters is necessary and sufficient.

For the indices estimated using PCA, only those components with eigenvalues > 1.0 were retained, whose relevance was confirmed by the scree plot, following the methodology described by den Reijer and their collaborators [67]. In the case of indices synthesized via MCA, in accordance with previous studies [68], dimensions with inertia of > 0.2 were retained after analysis of the inertia curve.

To examine the association between covariates and DPs, we used a two-stage analytical approach. First, we estimated the proportions and confidence intervals for the covariates for each DP. Next, the chi-square test was applied to compare the differences between proportions. The level of significance adopted was *P* < 0.05. For variables with more than two categories, a post hoc correction was applied using Bonferroni’s adjusted method.

To assess the association between DPs and their determinants, we used a discrete choice logit model. Initially, a crude analysis was performed to estimate prevalence ratios with 95% confidence intervals for each independent variable. At this stage, the variables of biological sex of the head of household and the index of diseases did not show a statistically significant association with the type of DP; however, in the adjusted analysis, all the independent variables were found to be significant at a 95% confidence level, except for the index of diseases.

It was decided to consider the adjusted model with all variables, given its epidemiological and theorical importance [45, 69, 70]; in addition, the Akaike information criterion (AIC) reported a value more than 5 points lower for the full model (with all variables; AIC = 5607) than the reduced model (without the variables biological sex of the head of household and the disease index; AIC = 5612), which indicates a better balance between fit and parsimony in the full model [71].

To compare magnitudes between covariates, following the recommendations of Norton and collaborators, multivariate logistic model average marginal effects (AMEs) were estimated [72]. AMEs quantify the change in the probability of adhering to a DP (vs other DP), for a one unit increase in each predictor, keeping the other variables at their mean values.

Multicollinearity was assessed using variance inflation factors (VIF), and all predictors yielded VIF < 3. On the other hand, adequate model fit was assessed by applying the Hosmer–Lemeshow test [73], which *P*-value was greater than 0.05, meaning that there are no significant differences between the observed frequencies and those predicted by the model. Data tabulation, cleaning, and the construction of indexes and indicators were carried out using SPSS v.29 (IBM Corp., Armonk, NY, USA), while Stata v.17 (StataCorp LLC, College Station, TX, USA) was used for prevalence estimation, calculation of factor loadings, and logistic regression analyses.

## Results

This study identified two distinct DPs (see Table 2). The first DP groups 63.26% of the population studied and was defined by higher factor loadings for refined foods. The foods typical of this pattern are characterized by high levels of sugars, saturated fats, and sodium, in addition to being hypercaloric. Among these, notable examples include ham, different types of jams, butter, cheese, chocolates, sweets, ice creams, and soft drinks. In this context, this pattern was named the rich in calories but poor in nutrients (RCPN) diet. The second pattern, on the other hand, presents a predominant consumption of fresh foods and a lower intake of refined cereals and added sugars. The reference foods of this second pattern are fresh fruits, whole grains (barley, corn, quinoa, and wheat), meat (chicken and beef), and a set of vegetables (beets and broccoli). Due to its peculiarities, this pattern was named the whole foods plant-based (WFP) diet.

**Table 2:** Factor-loading matrix of the two dietary patterns identified, based on Nutrition and Development Survey (2023) in Cochabamba, Bolivia

Variable^*^	Rich in calories but poor in nutrients	Whole foods plant-based
Snack/convenience foods	0.847	-
Sugar-sweetened beverages	0.815	-
Vegetables preparations	0.671	-
Preserved meats	0.612	0.360
High-fat dairy products	0.497	0.562
Refined cereals and grains	0.475	0.294
Sweetened fruit products	0.451	0.467
Infusions	0.238	0.559
Fruits	0.230	0.716
Meat	0.224	0.648
Vegetables	-	0.608
Cereals and grains	-	0.684
Dairy	-	0.609

^*^Only food groups having factor loadings > 0.200 are shown

Table 3 summarizes the general characteristics of the 4496 households included in the study. Overall, out of every ten households, six are in the peripheral areas, three are in the compact southern zone, and a smaller proportion is in the northern residential area. Heads of households were predominantly male, and 86.7% were between 17 and 65 years old. Half of the heads of households were engaged in non-manual activities, while 49.3% performed manual labor. More than half of the households were made up of three to four members, and more than two-thirds had a monthly food budget of less than US$259. Literacy was practically universal, with more than 99% of households being literate.

**Table 3:** Socio-demographic characteristics: total proportions and prevalence by dietary patterns of the population of Cochabamba Municipality, Bolivia

Indexes and indicators	Total (*n* = 4496)	Rich calorie but poor nutrients (*n* = 2844)	Whole foods plant based (*n* = 1652)	*P*-value
Zone of residence				
Northern residential^a^	13.3 (12.4, 14.4)	54.2 (50.2, 58.1)	45.8 (41.9–49.8)	0.005^*^
Northern periphery^ab^	28.8 (27.4, 30.1)	59.3 (56.6, 61.9)	40.7 (38.0–43.4)	
Compact south^b^	26.8 (25.5, 28.1)	63.3 (60.5, 65.9)	36.7 (34.0–39.5)	
Southern periphery^c^	30.9 (29.6, 32.3)	70.9 (68.5–73.2)	29.1 (26.8–31.5)	
Biological sex of the head of household				
Male	67.3 (65.8, 68.5)	63.4 (61.7, 65.2)	36.6 (34.8, 38.3)	0.704
Female	32.7 (31.4, 34.1)	62.9 (60.4, 65.3)	37.1 (34.7, 39.6)	
Age of head of household				
17–44^a^	47.4 (45.8, 48.8)	59.4 (57.3, 61.5)	40.6 (38.5, 42.7)	0.000^*^
45–65^b^	39.3 (37.8, 40.7)	64.8 (62.6, 67.0)	35.2 (33.0, 37.4)	
≥ 65^c^	13.3 (12.3, 14.3)	72.3 (68.8, 75.9)	27.7 (24.1, 31.2)	
Household size				
1–2 members^a^	26.9 (25.6, 28.2)	70.0 (67.4, 72.6)	30.0 (27.4, 32.6)	0.000^*^
3–4 members^b^	52.7 (51.2, 54.1)	59.9 (57.9, 61.9)	40.1 (38.1, 42.1)	
5 y + members^b^	20.3 (19.1, 21.5)	63.0 (59.9, 66.2)	37.0 (33.8, 40.1)	
Socioeconomic strata				
Low stratum^a^	12.6 (11.6, 13.6)	76.5 (73.0, 80.0)	23.5 (20.0, 27.0)	0.000^*^
Medium–low stratum^b^	43.8 (42.3, 45.2)	66.6 (64.5, 68.6)	33.4 (31.4, 35.5)	
Medium–high stratum^c^	43.5 (42.1, 45.0)	56.1 (53.9, 58.3)	43.9 (41.7, 46.1)	
Food expenditure ($US)				
< 259^a^	72.7 (71.3, 73.9)	65.6 (64.0, 67.3)	34.4 (32.7, 36.0)	0.000^*^
258–402^b^	19.4 (18.2, 20.6)	57.2 (53.9, 60.4)	42.8 (39.6, 46.1)	
> 402^b^	7.87 (7.12, 8.69)	56.2 (51.1, 61.4)	43.8 (38.6, 48.9)	
Socio-occupational stratum				
Manuals	49.3 (47.8, 50.8)	69.0 (67.1, 71.0)	31.0 (29.0, 32.9)	0.000^*^
Non-manual	50.7 (49.1, 52.1)	57.6 (55.6, 59.6)	42.4 (40.4, 44.4)	
Literacy rate				
Not literate	0.70 (0.40, 0.87)	81.5 (66.8, 96.1)	18.5 (3.9, 33.2)	0.049^*^
Literate	99.3 (99.1, 99.5)	63.1 (61.7, 64.6)	36.9 (35.4, 38.3)	
Diet index				
One diet	90.7 (89.8, 91.5)	62.2 (60.7, 63.7)	37.8 (36.3, 39.3)	0.000^*^
More than one diet	9.23 (8.41, 10.1)	74.0 (69.8, 78.2)	26.0 (21.8, 30.2)	
Migratory status index				
Migrant	25.0 (23.7, 26.3)	68.6 (65.9, 71.3)	31.4 (28.7, 34.1)	0.000^*^
Non-migrant	74.9 (73.6, 76.2)	61.5 (59.8, 63.1)	38.5 (36.9, 40.2)	
Unmet basic needs index				
Full access to basic services	68.2 (66.8, 69.6)	59.4 (57.7, 61.4)	40.6 (38.9, 42.3)	0.000^*^
Lack of at least one basic service	31.7 (30.3, 33.1)	71.5 (69.2, 73.9)	28.5 (26.1, 30.8)	
Housing construction quality index				
High housing construction quality	44.5 (54.0, 56.9)	58.0 (55.8, 60.2)	42.0 (39.8, 44.2)	0.000^*^
Low housing construction quality	55.4 (43.0, 45.9)	67.5 (65.6, 69.3)	32.5 (30.7, 34.4)	
Food culture index				
3 meals-breakfast (main meal)	42.4 (40.9, 43.8)	68.8 (66.7, 70.9)	31.2 (29.1, 33.3)	0.000^*^
< 3 meals-dinner (main meal)	57.6 (56.1, 59.0)	59.2 (57.3, 61.1)	40.8 (38.9, 42.7)	
The food purchasing space index				
Two places maximum	56.0 (54.5, 57.4)	66.8 (64.9, 68.6)	33.2 (31.4, 35.1)	0.000^*^
More than 2 places	44.0 (42.5, 45.4)	58.8 (56.6, 61.0)	41.2 (39.0, 43.4)	
Food preference index				
Flavor and appearance-processed foods	67.9 (66.5, 69.2)	65.0 (63.3, 66.6)	35.0 (33.4, 36.7)	0.001^*^
Weight control-natural foods	32.1 (30.7, 33.4)	59.7 (57.1, 62.2)	40.3 (37.8, 42.9)	
Index of diseases				
Chronic diseases	62.3 (60.9, 63.7)	63.7 (61.9, 65.5)	36.3 (34.5, 38.1)	0.419
Other diseases	37.7 (36.2, 39.0)	62.5 (60.2, 64.8)	37.5 (35.2, 39.8)	

All values are expressed as percentages (%)

^*^P-value calculated by the chi square test; *p*-value < 0.05. A post hoc correction was applied to variables with more than two categories, using the Bonferroni’s adjusted method. Different superscript letters indicate statistically significant differences in the prevalence among categories of each sociodemographic characteristic across dietary patterns. Categories sharing at least one letter do not differ significantly

Regarding socioeconomic status, 56.4% of the population was concentrated in the low and medium–low strata. Furthermore, more than one-third of the surveyed households did not have full access to basic services, and over half were constructed with low-quality materials. A quarter of the households corresponded to internal migrants (Table 3).

In terms of their food preferences, a large proportion stated that they followed a single eating pattern, ate fewer than three meals a day, bought their food in two different establishments, and preferred refined foods. Finally, a considerable proportion of households reported having at least one member with a chronic disease (Table 3).

Regarding prevalence by dietary pattern, significant differences were observed in all variables except for the biological sex of the head of household (*P* > 0.05) and the chronic disease index (*P* > 0.419). Initially, a growing gradient was observed from north to south, such that, as one moves from the northern residential area to the southern periphery, there is a progressive increase in the prevalence of the RCPN diet, accompanied by a parallel decrease in adherence to the WFP diet (*P* < 0.05). This was confirmed by finding significant contrasts between the northern residential area and the areas of the compact south and the southern periphery (Table 3).

The prevalence of the RCPN dietary pattern increased progressively with the age of the head of household (59.4% in the 17–44 age group, 64.8% in 45–65 age groups, and 72.3% in those ≥ 65 years old) (*P* < 0.05) and was more frequent in households with 1–2 members (*P* < 0.05), in the low and medium–low (*P* < 0.05) socioeconomic strata, households with a food budget of less than US$259 (*P* < 0.05), in manual workers (*P* < 0.05), illiterate heads of household (*P* < 0.05), and migrant households (*P* < 0.05). A higher prevalence of the RCPN pattern was also observed in homes with limited access to basic services (*P* < 0.05) and built with low-quality materials (*P* < 0.05). In terms of eating behavior, these households practice more than one diet (*P* < 0.05), consume three meals a day (*P* < 0.05), purchase their food from a maximum of two supply centers (*P* < 0.05), and showed preferences for processed foods based on their taste and appearance (*P* < 0.05) (Table 3).

In contrast, the WFP pattern predominated in households in the northern residential area, with young heads of households, households with 3–4 members, and more than 5 members, belonging to the medium–high socioeconomic stratum and with a higher food budget (*P* < 0.05). It was also more common among literate heads of households who performed non-manual activities, in non-migrant households, and in homes with full access to basic services and built with high-quality materials. In addition, those who adopted the WFP pattern consumed a maximum of two meals a day, purchased their food in more than two places, and selected their food with a focus on minimally processed items that would allow them to maintain an adequate weight (*P* < 0.05) (Table 3).

Overall, the RCPN pattern was associated with conditions with greater socioeconomic disadvantage, while the WFP pattern predominated in households with better socioeconomic conditions.

Table 4 presents the results of the adjusted logistic regression analysis, which show the associations between a set of demographic and socioeconomic factors and DP.

**Table 4:** Determinants of the rich calories and poor nutrient eating pattern: marginal effects, 95% CI, and *P*-values from multivariable logit analysis, municipality of Cochabamba, Bolivia

Indexes and indicators	Marginal effects	95%, CI	*P*-value
Zone of residence			
Northern residential	1	-	-
Northern periphery	0.05	(0.01; 0.09)	0.028
Compact south	0.07	(0.02; 0.11)	0.005
South periphery	0.08	(0.03; 0.13)	0.004
Biological sex of the head of household			
Female	1	-	-
Male	0.03	(0.00; 0.06)	0.024
Age of head of household			
17–44	1	-	-
45–65	0.06	(0.03; 0.09)	0.000
> 65	0.12	(0.08; 0.16)	0.000
Socioeconomic stratum			
Medium–high stratum	1	-	-
Medium–low stratum	0.04	(0.01; 0.08)	0.000
Low stratum	0.08	(0.03; 0.13)	0.001
Dietary index	0.1	(0.05; 0.14)	0.000
Migratory status index	0.06	(0.01; 0.10)	0.025
Food culture index	0.27	(0.19; 0.35)	0.000
Food preference index	0.17	(0.09; 0.26)	0.000
Disease index	0.22	(0.00; 0.44)	0.054
Household size			
1–2 members	1	-	-
3–4 members	− 0.09	(− 0.11; − 0.05)	0.000
5 and + members	− 0.07	(− 0.11; − 0.03)	0.001
Monthly food expenditure ($US)			
< 259	1	-	-
259–419	− 0.035	(− 0.07; 0.00)	0.055
≥ 420	− 0.045	(− 0.09; 0.01)	0.090
Socio-occupational stratum			
Manual	1	-	-
Non-manual	− 0.038	(− 0.07; − 0.01)	0.020
Literacy rate	− 0.23	(− 0.44; − 0.01)	0.039
Unmet basic needs index	− 0.16	(− 0.25; − 0.06)	0.002
Housing construction quality index	− 0.11	(− 0.18; − 0.04)	0.002
Food purchasing space index	− 0.1	(− 0.18; − 0.03)	0.009

Only in the case of the disease index a significant level of 10% was considered. This table only presents the marginal effects of the RCPN diet, since these are symmetrical (equal in magnitude but opposite in sign) to those of the WFP diet

First, the RCPN dietary pattern (*n* = 2844) showed a direct relationship with the area of residence and belonging to certain socioeconomic strata. Households living in the southern part of the municipality report a 7% higher probability of adopting this diet compared to those households located in the north. Likewise, a socioeconomic gradient is observed in DPs, where households in the low and medium–low strata showed higher probabilities (8% and 4%) of following a RCPN diet, compared to those in the medium–high stratum (Table 4).

In relation to biological sex, male-headed households report a 3% higher probability of adopting a RCPN diet than female-headed households. The relationship is similar in the case of the age of the head of households, since households with heads over 45 and 65 years of age report a 6% and 12% higher probability of adopting this DP, compared to households headed by young people (Table 4).

On the other hand, households composed of members who practice a single diet, are internal migrants, prepare three meals per day and choose their food guided by taste and appearance report greater adherence to the RCPN pattern. Then, each time these indexes increase by one unit, the probability that they adhere to this DP increases by 10, 6, 27, and 17 percentage points, respectively (Table 4).

It was also found that the disease index is statistically significant at a 90% confidence level and is positively associated with the RCPN DP, such that households with members who were diagnosed (either the head of household or their parents) with a cardio metabolic or cardiovascular disease report a 22% higher probability of adopting a RCPN diet, with respect to households following a WFP diet (Table 4).

In terms of material living conditions, the unsatisfied basic needs index and the housing construction quality index are negatively associated with the RCPN pattern, such that households with access to electricity, drinking water, sewerage, room exclusively for cooking, and construction with noble materials (walls with siding, wood floors, and tile roofs) report 16% and 11% lower probabilities of belonging to the group of households that adopt a RCPN diet (Table 4).

The adjusted model also revealed that household size, food expenditure (both inside and outside the home), literacy rate, socio-occupational stratum, and food shopping space index were inversely associated with the RCPN dietary pattern. The most notable impact corresponds to the literacy index, which shows that households with literate members report a 23% lower probability of adopting a RCPN diet (with respect to households with illiterate members). The shopping space index reveals that households that buy their food in more than two food centers are 10% less likely to adopt a RCPN diet (Table 4).

Regarding household size, it was found that, on average, households that follow an RCPN diet are composed of a reduced number of members. In the case of heads of household whose work activities are classified within the socio-occupational stratum of non-manual workers, they reported a 4% lower probability of following a RCPN diet. The trend is identical for food expenditures, where households with spending in the range of US$259–US$419 or more are 4% and 5% less likely to follow a RCPN diet than households with budget allocations of less than US$259 (Table 4).

## Discussion

This study identified two DPs in Cochabamba: RCPN and WFP. Key sociodemographic predictors included zone of residence, sex, age of head of households, socioeconomic stratum, dietary index, migratory status, food culture, food preference, disease, household size, food expenditure, socio-occupational stratum, literacy rate, unmet basic needs, housing construction quality, and food purchasing space.

To our knowledge, this is the first study to examine the relationships between DPs and demographic and socioeconomic variables, housing construction quality, food preferences, and prevalence of non-communicable diseases, based on a representative sample of Cochabamba’s urban and peri-urban population. In this regard, our study reveals the existence of a gap between the most advantaged and the most vulnerable population groups [74], highlighting the need to identify the determinants of these disparities to promote public policy interventions focused on the latter group.

In Latin America, several studies confirm the presence of an RCPN [75–77] and WFP [11, 16, 78, 79], as well as other DPs. However, in the Bolivian case, no studies were identified that characterized any DP; the few studies that addressed this topic were limited to describing the tendencies, perceptions, and composition of diets to determine ethnic and cultural differences in small population groups [15, 22, 80, 81].

Regarding the identified patterns, it was evident that both groups report a tendency towards homogenization in food consumption since both household types follow an RCPN pattern and those that practice a WFP diet consume ultra-processed foods together with minimally processed foods, with the differentiating category between groups being the frequency of consumption. This phenomenon is explained by the fact that Cochabamba, Bolivia, is going through a nutritional transition driven by more significant economic growth [82] and the coexistence of a traditional pattern (in our study called the WFP pattern) with an RCPN pattern, which in a synergistic effect established an incremental trend in the consumption of foods with a high degree of processing [83].

Cochabamba, once considered the breadbasket of Bolivia, is characterized by a segregated socio-spatial distribution [19]. Thus, urban poverty is concentrated in the peripheral areas of the municipality [20], mainly in the southern periphery, while Cochabamba’s economic elites live in the north and center of the city [81]. Although there are differences between areas, these are also evident among households in the peripheral regions, perhaps with greater intensity. On the same street, houses with two or more floors, built with good materials, and offering complete access to basic services, are alongside houses made of adobe, the vast majority of which lack water or sewage connections [20]. These spaces concentrate a large part of the population belonging to the low and medium–low socioeconomic stratum: households with a daily income per person of less than 4 and 10 US dollars (56.45%) and with a maximum formal education of secondary level [84].

These findings confirm that socioeconomically marginalized households have an 8% and 4% percentage points higher probability of adopting an RCPN diet, compared to the group of households belonging to the medium–high socioeconomic stratum, who prefer foods with minimal processing, with fruits and vegetables, as health protectors [16, 30, 85]. The greater adherence to an RCPN diet could be explained by the following: (a) households living in these areas do not encourage healthy eating behaviors, as these areas are full of fast-food restaurants [86] and neighborhood stores that offer mostly low-cost processed and ultra-processed foods [87] which facilitates a process of replacing local foods with high-energy–density foods [88]; and (b) the heads of these households report few years of formal education, which reduces the likelihood of these households adopting healthy behaviors, partly due to a lack of knowledge of the benefits of proper nutrition for health [89].

Regarding differences by biological sex, it was found that male-headed households had a higher probability of adopting an RCPN diet compared to female-headed households. This disparity may be attributed to differentiated gender roles and work dynamics in this region. In male-headed households, women tend to dedicate themselves to family care, spending much of their time on unpaid household chores [90]. This division of labor tends to prioritize the consumption of energy-dense foods, aimed at covering the caloric requirements of the head of household, who generally performs physically demanding work [91]. On the contrary, in female-headed households, women face a double burden: caring for their families and generating economic income (which is often poorly remunerated) [92]. This overload of responsibilities reduces their time and energy for cooking, which pushes them to choose foods that are quick to prepare. In addition, having greater autonomy in their food decisions, female heads of household tend to prioritize foods that are perceived as healthier, to take care of their children’s health [91]. It should be noted that, in Bolivia, the female head of household is usually recognized only in the absence of the husband—either by death, divorce, or abandonment [93].

Regarding migratory status, our study presents evidence that many migrant households are in less privileged areas and reports a marked adherence to the RCPN dietary pattern. This relationship could be explained by the fact that migrant households prefer ready-to-eat foods since to survive and guarantee the food security of their family; they require that both parents perform work activities, thus limiting their possibilities of preparing their food and at the same time increasing their spending on less nutritious foods outside the home [94].

Other determining factors of the identified DPs are the indices of unmet basic needs and the quality of housing construction. Lack of access to basic services and good housing construction quality (wooden floors, cladding walls, and tiled roofs) is considered an indicator of economic poverty [95], and is negatively associated with the RCPN pattern, such that marginalized households are 16% and 11% more likely to adopt this DP compared to more advantaged households [74].

These factors constitute an important finding of our study since, in the context of studies related to the topic of DPs in Latin America; these are usually developed with data from secondary sources, which mostly focus their attention on the identification of causal relationships between socioeconomic and sociodemographic factors at the individual level, leaving aside aspects related to the spatial dimension, in which these households coexist [96, 97].

The healthy food preference factor replicates the evidence presented by Newby and Tucker [98], who highlight the importance of food for weight control. Our study found that heads of households concerned about maintaining an adequate body weight by consuming foods that allow for it report a greater likelihood of adopting a WFP diet. The diet index and the food culture index report a behavior similar to the previous one; so, households that at one time followed some diet (vegan, keto, among others) and those who consider breakfast as the main meal and make three food preparations daily show a greater probability of following a WFP diet. In the case of the food purchasing space index, it was evident that households that buy their food in a few spaces report a greater adherence to the RCPN pattern. This phenomenon could be explained by the fact that the food supply providers of large supermarket chains and popular markets usually are the same [99].

Finally, the disease index reports results that are consistent with the literature. There was evidence of a positive association between the higher prevalence of chronic cardiovascular and cardiometabolic diseases and the RCPN pattern [30, 83, 100, 101]. An important aspect to consider is that our NCD index captures the intergenerational transmission of chronic diseases, which opens a vein for the development of long-term research programs that seek to elucidate the hypothesis that the adoption of a DP is transmitted from generation to generation and, therefore, also the probability of developing an NCD that the parents have previously reported.

The results presented here are limited by the incomplete capture of seasonal variation in diet, as data were collected over 6 months (between October and December 2022 and January and March 2023). This limitation posed the problem of overestimating the results since, without longitudinal data, the possibilities of making comparisons are limited. Another aspect that could have affected the findings is language and geographic access to specific regions. In certain areas, primarily in the outskirts, interviewees had difficulty understanding Spanish, which may have led to confusion and omissions in recording their responses. Additionally, locating the heads of these households was challenging, despite efforts to visit them on weekends. To address this limitation, it was necessary to re-administer the surveys with bilingual staff who also lived near the areas of interest. Finally, the surveys were conducted by field staff with different levels of experience, which could have influenced the consistency of data collection, introducing variability in respondents’ responses due to differences in the way they interacted with them or in the interpretation of the questions.

Despite the limitations of this study, the large sample size and the rigorous quality control in data collection, which in some cases involved re-collecting the survey, could make our results generalizable to the context of Cochabamba and Bolivia.

## Conclusions

Two DPs were identified among the urban population of the Municipality of Cochabamba. The RCPN pattern is more prevalent in households belonging to the low and medium–low socioeconomic strata of the peripheral areas, while the WFP pattern is associated with better socioeconomic conditions.

It was found that marginalized households are the most vulnerable to chronic health problems, as their diet consists of less nutritious foods, typical of an RCPN diet. Although Bolivia seeks to guarantee household food security with fuel subsidies and a package of measures to keep inflation under control, these do not solve the problem of poverty, which is the basis of many social problems, and the fact is that marginalized households, lacking resources, time, and guidance, cannot avoid the RCPN diet [18]. The identification of this last DP, which is composed exclusively of dairy products, infusions, sugary drinks, and high-calorie foods, poses a potential health risk.

This study analyzed how socioeconomic and demographic characteristics create the scenario for a nutritional and food transition marked by a greater consumption of processed and ultra-processed foods, which, in the second stage, would probably lead to an exponential increase in NCDs. This study may contribute to the field of nutritional epidemiology and policymakers by offering possible opportunities for actions to prevent the harmful effects of the nutritional transition in the Cochabamba population.

## Supplementary Information

Supplementary material 1. Table S1. Foods from the food consumption frequency survey, according to degree of processing, Cochabamba, Bolivia, contains the complete list of foods included in the food frequency questionnaire. Table S2. Nutrient intake reproducibility between FFQ and R24, presents reproducibility and concordance indicators.

Supplementary material 2. Includes the full version of the Nutrition and Development Survey. The document is presented in both Spanish and English.

## Data Availability

The anonymized, aggregated dataset and analytic codes used to reproduce our analysis are available in the Zenodo unrestricted repository (DOI: 10.5281/zenodo.17295428).

## Abbreviations

DP: Dietary pattern
RCPN: Rich calorie and poor nutrient diet
WFP: Whole food plant-based diet
NCD: Non-communicable diseases
FFQ: Food Frequency Questionnaire
R24: 24-Hour recall
ICC: Intraclass correlation coefficient
CCC: Concordance correlation coefficient
HCA: Hierarchical cluster analysis
PCA: Principal component analysis
MCA: Multiple correspondence analysis
LMICs: Low- and middle-income countries
AIC: Akaike information criterion
NDS: Nutrition and Development Survey
PSU: Primary sample unit
