# Supplementary material for: Dietary patterns and socioeconomic disadvantage: an analysis of food consumption patterns and their determinants in Cochabamba, Bolivia
Source: BMC Glob Public Health. 2025 Nov 20;3:104. doi: 10.1186/s44263-025-00221-2 (PMC12632106; doi:10.1186/s44263-025-00221-2)
Supplement: Supplementary file 1 — Supplementary material 1. Table S1. Foods from the food consumption frequency survey, according to degree of processing, Cochabamba, Bolivia, contains the complete list of foods included in the food frequency questionnaire. Table S2. Nutrient intake reproducibility between FFQ and R24, presents reproducibility and concordance indicators. [file 44263_2025_221_MOESM1_ESM.docx]

**Table S1** Foods from the food consumption frequency survey, according to degree of processing, Cochabamba, Bolivia.

| **NOVA Classification** | **Foods** | | |
| --- | --- | --- | --- |
| Minimally processed foods | Potato | Orange | Rice |
|  | Sweet potato | Mandarin orange | Oats |
|  | Oca | Lemon | Barley |
|  | Tomato | Guava | Flaxseed |
|  | Locoto pepper | Papaya | Cañahua |
|  | Onion | Pomegranate | Honey |
|  | Carrot | Avocado | Beef |
|  | Broccoli | Grape | Chicken |
|  | Lettuce | Pacay | Fish |
|  | Beetroot | Pear | Pork |
|  | Stuffing cucumber | Kiwi | Llama meat |
|  | Cucumber | Egg | Rabbit meat |
|  | Garlic | Quail egg | Duck meat |
|  | Cilantro | Pasteurized milk | Lentils |
|  | Parsley | Ground cereals | Peanuts |
|  | Plantain | Dry cereals | Chickpeas |
|  | Fig | Dried fruits | Chamomile |
|  | Pineapple | Wheat | Coca leaves |
|  | Watermelon | Quinoa | - |
|  | Apple | Corn | - |
| Processed culinary ingredients | Sunflower oil | Cane sugar | Cornstarch |
|  | Soybean oil | Salt | Tea |
|  | Olive oil | Vanilla extract | - |
|  | Pepper | Coconut sugar | - |
| Processed foods | Canned vegetables | Jams | Instant coffee |
|  | Fruit in syrup | Canned fish | Butter |
|  | Bread | Canned corn | Dried fruit |
|  | Cheese | Beer | Soy sauce |
|  | Noodle | Ham | - |
| Ultra-processed foods | Soft drinks | Potato chips | Condensed milk |
|  | Artificial fruit juices | Sugary boxed cereals | Chicken nuggets |
|  | Ice cream | Packaged sweet rolls | Pre-cooked pizza |
|  | Chocolate | Flavored yogurt | Tacos |
|  | Candy | Instant soups | Nachos |
|  | Sweets | Reconstituted beef | Sausage |
|  | Cookies | Fortified flour | - |

**Table S2** Nutrient intake reproducibility between FFQ and R24

| **Energy and nutrients** | **r^[[1]](#footnote-1)^** | **ICC Adjusted^[[2]](#footnote-2)^** | **CCC^[[3]](#footnote-3)^** |  |
| --- | --- | --- | --- | --- |
|  |  |  |  |  |
| Energy (Kcal) | 0.7566** | - | 0.727 [0.636, 0.818] |  |
| Proteins (g) | 0.6951** | 0.689 [0.568, 0.780] | 0.678 [0.560, 0.770] |  |
| Fats (g) | 0.5637** | 0.563 [0.412, 0.685] | 0.545 [0.394, 0.667] |  |
| Carbohydrates (g) | 0.6755** | 0.667 [0.540, 0.764] | 0.615 [0.489, 0.716] |  |
| Ca (mg) | 0.3833* | 0.374 [0.191, 0.533] | 0.302 [0.151, 0.439] |  |
| P (mg) | 0.4701** | 0.470 [0.300, 0.611] | 0.460 [0.293, 0.600] |  |
| Na (mg) | 0.3206** | 0.316 [0.126, 0.483] | 0.299 [0.121, 0.458] |  |
| K (mg) | 0.1418 | 0.118 [-0.082, 0.308] | 0.041 [-0.017, 0.099] |  |
| Fe (mg) | 0.6984* | 0.691 [0.561, 0.787] | 0.686 [0.558, 0.781] |  |
| Se (mg) | 0.5922** | 0.527 [0.363, 0.658] | 0,313 [0,209, 0,409] |  |
| Tiamin (mg) | 0.4478** | 0.466 [0.272, 0.591] | 0.355 [0.207, 0.487] |  |
| Riboflavin (mg) | 0.4410** | 0.433 [0.258, 0.581] | 0.423 [0.254, 0.567] |  |
| Niacin (mg) | 0.7037** | 0.692 [0.573, 0.783] | 0.636 [0.517, 0.732] |  |
| Folic Acid (mg) | 0.2869** | 0.164 [-0.036, 0.352] | 0.035 [0.010, 0.061] |  |
| Vitamin B12 (mg) | 0.7692** | 0.769 [0.664, 0.844] | 0.766 [0.662. 0.842] |  |
| Vitamin C (mg) | 0.4378** | 0.423 [0.246, 0.573] | 0.392 [0.231, 0.532] |  |
| Vitamin A (mg) | 0,2235* | 0.149 [-0.050, 0.336] | 0.026 [0.002, 0.049] |  |

1. Pearson’s correlation coefficient, level of statistical significance: *P<0.05, **P<0.01 [↑](#footnote-ref-1)
2. ICC intraclass correlation coefficient adjusted [↑](#footnote-ref-2)
3. CCC Lin’s concordance correlation coefficient [↑](#footnote-ref-3)
