# Supplementary material for: Dietary patterns and socioeconomic disadvantage: an analysis of food consumption patterns and their determinants in Cochabamba, Bolivia
Source: BMC Glob Public Health. 2025 Nov 20;3:104. doi: 10.1186/s44263-025-00221-2 (PMC12632106; doi:10.1186/s44263-025-00221-2)
Supplement: Supplementary file 2 — Supplementary material 2. Includes the full version of the Nutrition and Development Survey. The document is presented in both Spanish and English. [file 44263_2025_221_MOESM2_ESM.pdf]

UNIVERSIDAD MAYOR DE SAN SIMÓN
CENTRO DE PLANIFICACIÓN Y GESTIÓN
ENCUESTA DE NUTRICIÓN Y DESARROLLO

Programa de doctorado en ciencias sociales y humanidades

UMSS

CEPLAG
Centro de Planificación y Gestión

Sweden
Sverige

UNIVERSIDAD
MAYOR DE SAN SIMÓN
Ciencia y Conocimiento desde 1832

UNIVERSIDAD MAYOR DE SAN SIMÓN
CENTRO DE PLANIFICACIÓN Y GESTIÓN
ENCUESTA DE NUTRICIÓN Y DESARROLLO

Número de boleta: ....

PRESENTACIÓN

El objetivo de esta encuesta es contar con información actualizada acerca de las condiciones socioeconómicas, culturales y biogeográficas de la población del municipio de Cochabamba, relacionadas con el estado de nutrición y salud (y sus potencialidades de mejoramiento), así como su vinculación con otras variables que influyen en la calidad de vida de su población.

CONFIDENCIALIDAD

Sus respuestas serán tratadas de manera confidencial y agrupada con las de otras personas que están colaborando en el estudio. Muchas gracias.

MUNICIPIO	DISTRITO	ZONA	MANZANO	VIVIENDA	HOGAR	NRO DE HOGARES EN LA VIVIENDA	OBSERVACIONES

COD ENCUESTADOR	
COD SUPERVISOR	
COD CODIFICADOR	
COD DIGITADOR	
COD BOLETA	
FECHA	

A. CARACTERISTICAS SOCIO-DEMOGRAFICAS

A5. ¿Sabe leer y escribir?	A6. Cuál es su estado civil:<br>1. Casado<br>2. Conviviente<br>3. Separado<br>4. Divorciado<br>5. Viudo<br>6. Soltero	A7. ¿Cuál fue el nivel y curso más alto de instrucción que alcanzo?<br>1. Primaria<br>2. Secundaria<br>3. Técnico medio<br>4. Técnico superior<br>5. Instituto militar<br>6. Instituto policial<br>7. Normal<br>8. Universidad<br>9. Postgrado diplomado<br>10. Postgrado maestría<br>11. Postgrado doctorado<br>12. Ninguno	A8. El establecimiento donde estudia (o el último donde estudió) es (era):	A9. Durante este año ¿se inscribió en algún curso, grado de educación escolar, alternativa, superior o postgrado?	A10. ¿Qué idiomas habla? (respuesta múltiple)<br>1. Castellano<br>2. Aymará<br>3. Quechua<br>4. Guaraní<br>5. Otro (especifique)<br>6. Extranjero (ingles, francés, alemán, portugués, etc.)
1. Sí ☐<br>2. No ☐		Curso: ________<br>Nivel: ________	1. Fiscal ☐<br>2. Particular ☐<br>3. No asistió ☐	1. Sí ☐<br>2. No ☐	
1. Sí ☐<br>2. No ☐		Curso: ________<br>Nivel: ________	1. Fiscal ☐<br>2. Particular ☐<br>3. No asistió ☐	1. Sí ☐<br>2. No ☐	
1. Sí ☐<br>2. No ☐		Curso: ________<br>Nivel: ________	1. Fiscal ☐<br>2. Particular ☐<br>3. No asistió ☐	1. Sí ☐<br>2. No ☐	
1. Sí ☐<br>2. No ☐		Curso: ________<br>Nivel: ________	1. Fiscal ☐<br>2. Particular ☐<br>3. No asistió ☐	1. Sí ☐<br>2. No ☐	
1. Sí ☐<br>2. No ☐		Curso: ________<br>Nivel: ________	1. Fiscal ☐<br>2. Particular ☐<br>3. No asistió ☐	1. Sí ☐<br>2. No ☐	
1. Sí ☐<br>2. No ☐		Curso: ________<br>Nivel: ________	1. Fiscal ☐<br>2. Particular ☐<br>3. No asistió ☐	1. Sí ☐<br>2. No ☐	
1. Sí ☐<br>2. No ☐		Curso: ________<br>Nivel: ________	1. Fiscal ☐<br>2. Particular ☐<br>3. No asistió ☐	1. Sí ☐<br>2. No ☐	

B. MIGRACIÓN

N° persona	**B1. ¿Dónde nació?**<br>1. País<br>2. Provincia<br>3. Municipio<br>4. Departamento<br>5. Localidad<br>6. Distrito	**B2. ¿Dónde vivía hace 5 años (2018)?**<br>1. Aquí → **B4**<br>2. En otro lugar → **B3**<br>2a. País<br>2b. Provincia<br>2c. Municipio<br>2d. Departamento<br>2e. Localidad<br>2f. Distrito	**B3. ¿Cuál fue la razón por la que dejo ese lugar?**<br>1.1. Búsqueda de trabajo<br>1.2. Traslado de trabajo<br>1.3. Educación<br>1.4. Salud<br>1.5. Razón familiar<br>1.6. Otra razón (especifique)	**B4. ¿Hace cuánto tiempo (años) vive o ha retornado a este lugar?**	**B5. ¿Dónde vivía inmediatamente antes de su lugar de residencia actual?**<br>1. País<br>2. Provincia<br>3. Municipio<br>4. Departamento<br>5. Localidad<br>6. Distrito	**B6. ¿Dónde viven sus padres y abuelos?**<br>1. País<br>2. Provincia<br>3. Municipio<br>4. Departamento<br>5. Localidad<br>6. Distrito	**B7. ¿Por qué escogió usted o su familia ese lugar?**<br>1. Tenía parientes o conocidos<br>2. Oportunidades de educación<br>3. Oportunidades de trabajo<br>4. Trabajo antes en el lugar<br>5. Mejorar condiciones de vida<br>6. Tenía trabajo asegurado<br>7. Por razones familiares<br>8. Otros (especifique)	**B8. ¿Tiene algún familiar en el extranjero?**<br>1. Sí → **B8**<br>2. No → **Sección C**	**B9. ¿En qué país? Especifique el año de salida**<br>1. País<br>2. Año de salida	**B10. ¿Cuál es su relación de parentesco?**<br>1. Jefe (a) de Hogar<br>2. Esposo (a)<br>3. Conviviente<br>4. Hijo (a)<br>5. Otro pariente
**1**	1<br>2<br>3<br>4<br>5	2a<br>2b<br>2c<br>2d<br>2e			1<br>2<br>3<br>4<br>5	1<br>2<br>3<br>4<br>5		1. Sí ☐<br>2. No ☐	1<br>2	
**2**	1<br>2<br>3<br>4<br>5	2a<br>2b<br>2c<br>2d<br>2e			1<br>2<br>3<br>4<br>5	1<br>2<br>3<br>4<br>5		1. Sí ☐<br>2. No ☐	1<br>2	
**3**	1<br>2<br>3<br>4<br>5	2a<br>2b<br>2c<br>2d<br>2e			1<br>2<br>3<br>4<br>5	1<br>2<br>3<br>4<br>5		1. Sí ☐<br>2. No ☐	1<br>2	
**4**	1<br>2<br>3<br>4<br>5	2a<br>2b<br>2c<br>2d<br>2e			1<br>2<br>3<br>4<br>5	1<br>2<br>3<br>4<br>5		1. Sí ☐<br>2. No ☐	1<br>2	
**5**	1<br>2<br>3<br>4<br>5	2a<br>2b<br>2c<br>2d<br>2e			1<br>2<br>3<br>4<br>5	1<br>2<br>3<br>4<br>5		1. Sí ☐<br>2. No ☐	1<br>2	
**6**	1<br>2<br>3<br>4<br>5	2a<br>2b<br>2c<br>2d<br>2e			1<br>2<br>3<br>4<br>5	1<br>2<br>3<br>4<br>5		1. Sí ☐<br>2. No ☐	1<br>2	
**7**	1<br>2<br>3<br>4<br>5	2a<br>2b<br>2c<br>2d<br>2e			1<br>2<br>3<br>4<br>5	1<br>2<br>3<br>4<br>5		1. Sí ☐<br>2. No ☐	1<br>2	

C. EMPLEO (PARA TODAS LAS PERSONAS DE 5 AÑOS O MAS)

N° persona	**C1. ¿Cuál fue su principal actividad laboral durante la semana pasada?**<br><br>1. Trabajó<br>2. No trabajó, pero tenía vacación<br>3. Buscó trabajo (cesante)<br>4. Buscó trabajo por 1° vez<br>5. Labores de casa y trabajó<br>(1–5) → **C4**<br><br>6. Sólo labores de casa<br>7. Sólo estudió<br>8. Es jubilado o rentista<br>9. Otro (especifique)<br>(6–9) → **C2**	**C2. ¿Realizó alguna actividad que aportó ingresos a su familia y a usted mismo?**<br>1. Sí<br>2. No	**C3. ¿Hizo algo para encontrar trabajo?**<br>1. Consulto con empleadores.<br>2. Asistió a una entrevista de trabajo<br>3. Contesto anuncios<br>4. Consulto en internet<br>5. Consulto con amigos, parientes o personas conocidas<br>6. Realizo consultas en periódico<br>7. Coloco su hoja de vida en redes sociales<br>8. No hizo nada → **D1**	**C4. ¿Cuál fue su principal ocupación la semana pasada? (o su última ocupación si estuvo desocupado)**<br><br>26301 Economistas<br>42206 Recepcionista<br>51200 Cocineros<br>95102 Lustrabotas<br>91101 Trabajadoras del hogar<br><br>***Utilice los códigos COB***	**C5. En su ocupación principal usted trabaja como:**<br>1. Empleado<br>2. Obrero<br>3. Trabajador cuenta propia<br>4. TCP subordinado a una sola empresa o capital<br>5. Familiar sin remuneración<br>6. Patrón o empleador<br>7. Profesional independiente<br>8. Empleado/a doméstico	**C6. ¿A qué se dedicaba el establecimiento en el cual trabaja (o en el que trabajó por última vez)?**<br><br>47921 Vendedor ambulante<br>49222 Transporte en taxi, radiotaxi y mototaxi<br><br>***Utilice los códigos CAEB***	**C7. ¿Por qué dejo su anterior ocupación?**<br>1. Finalización de contrato<br>2. Recorte de personal<br>3. Encontró un mejor empleo<br>4. Otro (especifique)	**C8. En su ocupación anterior usted trabajaba como:**<br>1. Empleado<br>2. Obrero<br>3. Trabajador cuenta propia<br>4. TCP subordinado a una sola empresa o capital<br>5. Familiar sin remuneración<br>6. Patrón o empleador<br>7. Profesional independiente<br>8. Empleado/a doméstico	**C9. ¿En qué rubro le gustaría desempeñar funciones?**<br><br>47921 Vendedor ambulante<br>49222 Transporte en taxi, radiotaxi y mototaxi<br><br>***Utilice los códigos CAEB***
**1**									
**2**									
**3**									
**4**									
**5**									
**6**									
**7**									

C. EMPLEO (SOLO OCUPADOS Y CESANTES)

N° persona	**C10. Su trabajo es (era):**<br>1. Permanente<br>2. Temporal<br>3. Ocasional	**C11. ¿Tiene (tenía) contrato de trabajo?**<br>1. Sí, con duración de 3 meses<br>2. Sí, a plazo fijo<br>3. Sí, de duración indefinida<br>4. No tiene (tenía) contrato	**C12. ¿Qué recibe (recibía) usted en el establecimiento donde trabaja (trabajaba)? (puede marcar más de una opción):**<br>1. Aguinaldo<br>2. Vacaciones<br>3. Atención de seguridad social<br>4. Indemnización<br>5. Ninguno de los anteriores	**C13. El ingreso que obtiene (Obtenía) por su trabajo es (era):**<br>1. Sueldo fijo<br>2. Sueldo fijo y porcentaje<br>3. Destajo o porcentaje<br>4. Jornal<br>5. Ingreso por trabajo independiente<br>6. NO recibe remuneración en dinero<br>7. Otros (especifique)	**C14. Normalmente ¿cuántos días y horas trabaja (trabajaba) a la semana?**		**C15. ¿Cuánto gana normalmente?**<br>(Pregunte por el ingreso neto o disponible)		**C16. Aproximadamente, ¿cuántas personas trabajaban en el establecimiento durante la última semana? (Incluyéndolo a usted)**
					**Días**	**Horas/día**	Frecuencia<br>1. Por día<br>2. Por semana<br>3. Por mes<br>4. Por año	**Monto (Bs.-)**	
1									
2									
3									
4									
5									
6									
7									

D. ANTECEDENTES FAMILIARES Y SALUD

N° persona	**D1. ¿Su familia tiene acceso a algún sistema de seguro de salud?**<br>1. Caja Nacional<br>2. Seguro privado<br>3. Otro (especifique)<br>4. No tiene	**D2. Principalmente, ¿Dónde acude en caso de enfermedad?**<br>1. Hospital<br>2. Clínica Privada<br>3. Seguro Médico<br>4. Consultorio Privado<br>5. Posta o Centro de Salud<br>6. Yatiri<br>7. Jampiq<br>8. Curandero<br>9. Compra en la farmacia sin receta<br>10. Medicina casera<br>11. Otro (especifique)	**D3. ¿Cuáles son los problemas de salud más frecuentes en su familia?**<br>1. Resfrío<br>2. Tos<br>3. Diarrea<br>4. Infecciones<br>5. Otros (especifique)	**D4. ¿Su médico le diagnosticó alguna de las siguientes enfermedades?**<br>1. Enfermedad isquémica<br>2. Diabetes mellitus<br>3. Diabetes insípida<br>4. Hipertensión<br>5. Osteoporosis<br>6. Obesidad<br>7. Cáncer<br>8. Hígado graso<br>9. Enfermedad renal crónica<br>10. Problema de tiroides<br>11. Síndrome de mala absorción<br>12. Enfermedades respiratorias<br>13. Enfermedad autoinmune ¿Cuál?<br>14. Ninguna enfermedad<br>15. Otro (especifique)	**D5. ¿Sus padres sufren o sufrieron algunas de las siguientes enfermedades?**<br>1. Enfermedad isquémica<br>2. Diabetes mellitus<br>3. Diabetes insípida<br>4. Hipertensión<br>5. Osteoporosis<br>6. Obesidad<br>7. Cáncer<br>8. Hígado graso<br>9. Enfermedad renal crónica<br>10. Problema de tiroides<br>11. Síndrome de mala absorción<br>12. Enfermedades respiratorias<br>13. Enfermedad autoinmune ¿Cuál?<br>14. Ninguna enfermedad<br>15. Otro (especifique)	**D6. ¿Sigue algún tipo de dieta especial?**<br>1. Sí<br>2. No → **E1**<br>3. No sabe → **E1**	**D6a. ¿En qué consiste su dieta? (respuesta múltiple)**<br>1. Vegetariana<br>2. Vegana<br>3. Baja en calorías<br>4. Baja en grasas<br>5. Baja en carbohidratos<br>6. Baja en proteínas animales<br>7. Alta en grasas o keto<br>8. Alta en proteínas<br>9. Libre de gluten<br>10. Liquida o blanda<br>11. Baja en sodio<br>12. Omite alim. Procesados<br>13. Omite gaseosas y azúcar<br>14. Omite lácteos<br>15. Omite harinas blan.<br>16. Omite grasas transgénicas<br>17. Otra (especifique)	**D7. ¿Desde cuándo sigue esta dieta? (Semanas)**	**D8. ¿Quién le prescribió o recomendó la dieta?**<br>1. Médico<br>2. Familiar<br>3. Amigo<br>4. Vecino<br>5. Curandero<br>6. Internet<br>7. Nadie<br>8. Otro (especifique)
1									
2									
3									
4									
5									
6									
7									

E. FECUNDIDAD Y MORTALIDAD INFANTIL: SOLO PARA MUJERES EN EDAD FERTIL DE 12 AÑOS Y MAS

N° persona	E1. ¿Cuántos hijos o hijas nacidos vivos ha tenido?<br>_____________hij@s	E2. De estos, ¿cuántos viven?	E3. ¿En qué mes y año nació su último hijo o hija nacido vivo?		E4. ¿Este último hijo vive?<br>2. No → **E1**<br>3. No sabe → **E1**	E5. ¿En qué fecha murió?		E6. ¿Quién atendió su último parto?<br>1. Médico<br>2. Enfermera<br>3. Partera<br>4. Familiar<br>5. Otro (especifique)
			**Mes**	**Año**		**Mes**	**Año**	
1								
2								
3								
4								
5								
6								
7								

F. COMPORTAMIENTO Y PREFERENCIAS DE CONSUMO FUERA DEL HOGAR (Miembros del hogar)

N° persona	**F1. Cuando come fuera de casa, ¿suele optar, con mayor frecuencia, por?:**<br>1. Comida casera<br>2. Ensaladas<br>3. Comida tradicional<br>4. Hamburguesas<br>5. Carnes a la parrilla<br>6. Frituras<br>7. Empanadas<br>8. Tortas, masas, helados<br>9. Otro (especifique)	**F1a. ¿Dónde?**<br>1. Vendedor ambulante<br>2. Caseta de mercado<br>3. Restaurante<br>4. Comedor universitario<br>5. Kiosco<br>6. Casa de un amigo (a)<br>7. Otro (especifique)	**F1b. ¿Con quién?**<br>1. Con amigos<br>2. Con mi pareja<br>3. Con mis compañeros de trabajo<br>4. Come solo (a)<br>5. Otro (especifique)	**F2. Indique los tipos de alimentos que más consume (ordinal).**<br>1 Alimentos naturales.<br>2. Mínimamente procesados.<br>3. Ingredientes culinarios.<br>4. Alimentos procesados.<br>5. Alimentos ultra procesados.	**F3. De las siguientes opciones, ¿cuáles son los atributos que considera más importantes en sus alimentos?**<br><br>**Determine en orden de importancia.**<br><br>a) Sean Nutritivos<br>b) Sean ricos en proteínas<br>c) Sean ricos en fibra	**F4. De las siguientes opciones, ¿cuáles son los atributos que considera más importantes en sus alimentos?**<br><br>**Determine en orden de importancia.**<br><br>a) Sean bajos en calorías<br>b) Sean bajos en azúcar<br>c) Sean bajos en grasas	**F5. De las siguientes opciones, ¿cuáles son los atributos que considera más importantes en sus alimentos?**<br>**Determine en orden de importancia.**<br><br>a) Sean de un lugar o marca conocida<br>b) Tenga buen sabor<br>c) Tenga buena presentación<br>d) Tenga un precio bajo<br>e) Me ayuden a controlar mi peso<br>f) Contengan ingredientes naturales	**F6. De las siguientes opciones, ¿cuál es tu favorita?**<br>1. Hamburguesa<br>2. Pizza<br>3. Pollo frito<br>4. Salchipapa<br>5. Carnes a la parrilla<br>6. Otro (especifique)	**F7. ¿Qué alimentos prefiere no consumir?**
1				1) ......<br>2) ......<br>3)	1) ......<br>2)	1) ......<br>2)	1) ......<br>2)		
2				1) ......<br>2) ......<br>3)	1) ......<br>2)	1) ......<br>2)	1) ......<br>2)		
3				1) ......<br>2) ......<br>3)	1) ......<br>2)	1) ......<br>2)	1) ......<br>2)		
4				1) ......<br>2) ......<br>3)	1) ......<br>2)	1) ......<br>2)	1) ......<br>2)		
5				1) ......<br>2) ......<br>3)	1) ......<br>2)	1) ......<br>2)	1) ......<br>2)		
6				1) ......<br>2) ......<br>3)	1) ......<br>2)	1) ......<br>2)	1) ......<br>2)		
7				1) ......<br>2) ......<br>3)	1) ......<br>2)	1) ......<br>2)	1) ......<br>2)		

UNIDAD DE ANÁLISIS: HOGAR

G. MORTALIDAD

G1. De enero de 2019 a la fecha, ¿murió alguna persona que vivía con ustedes en este hogar?

1. Sí ☐ **¿Cuántas personas?** ____________ → **G2.**
2. No ☐ → **Sección H.**

N° persona	G2. Nombre de la persona.	G3. Fecha de defunción		G4. ¿Qué edad tenía al morir?	G5. La persona era: 1. Hombre 2. Mujer	G6. ¿Se emitió certificado de defunción por el SERECI? 1. Sí 2. No	G7. ¿Murió a causa del COVID-19? 1. Sí 2. No	G8. Murió por alguna causa relacionada con: (puede marcar más de una opción) 1. Enfermedad isquémica del corazón 2. Diabetes 3. Hipertensión 4. Osteoporosis 5. Obesidad 6. Desnutrición 7. Cáncer 8. Enfermedad crónica del hígado 9. Enfermedad renal crónica 10. Problema de tiroides 11. Síndrome de mala absorción 12. Enfermedades respiratorias 13. Suicidio 14. Causa natural 15. Otro (especificar)
		Mes	Año					
1								
2								
3								
4								
5								
6								
7								

H. VIVIENDA (Solo para el principal informante)

H1. Tipo de vivienda:
1. Casa independiente ☐
2. Departamento ☐
3. Habitación (es) suelta (s) en casa de vecindad ☐
4. Choza, pahuichi ☐
5. Vivienda improvisada ☐
6. Local destinado a vivienda ☐
7. Otra (especifique) ________________ ☐

H2. Paredes
1. Adobe con revestimiento ☐
2. Adobe sin revestimiento ☐
3. Ladrillo ☐
4. Piedra ☐
5. Bloque de cemento ☐
6. Caña, palma, otras ☐
7. Otro (especifique) ________________ ☐

H3. Piso
1. Madera ☐
2. Mosaico o baldosas ☐
3. Ladrillo ☐
4. Cemento ☐
5. Tierra ☐
6. Otro (especifique) ________________ ☐

H4. Techo
1. Teja cerámica ☐
2. Teja cemento/fibrocemento ☐
3. Loza hormigón ☐
4. Calamina ☐
5. Teja de barro ☐
6. Paja, caña o palma ☐
7. Otro (especifique) ________________ ☐

H5. Cielo Raso
1. Bloques de yeso ☐
2. Estucado ☐
3. Tumbado (tela) ☐
4. No tiene ☐
5. Otro (especifique) ________________ ☐

H6. Esta vivienda es:
1. Propia ya pagada → **H9** ☐
2. Propia y la está pagando → **H9** ☐
3. Alquilada → **H8** ☐
4. Anticrético → **H8** ☐
5. Prestada o cedida por servicio ☐
6. Otra forma de tenencia (especifique) ________________ ☐

H7. Indique el número de habitaciones SOLO para dormir:
__________ Habitaciones

H7a. Cuántas habitaciones tiene en total sin contar baño y cocina
__________ Habitaciones

H8. ¿Cuánto paga mensualmente por concepto de alquileres o amortizaciones? (Para contrato anticrético, regístrese, además, el monto fijo del contrato)

Bs.- __________
Alquiler ☐
Amortización ☐

Bs.- __________
Monto fijo ☐
Anticrético ☐

H9. Cómo obtuvo esta Vivienda
1. La compro hecha ☐
2. La mandó a construir ☐
3. La construyó poco a poco ☐
4. Herencia o donación ☐
5. Crédito ☐
6. Remesa ☐
7. Otro (Especifique) ________________ ☐

H10. ¿En qué año adquirió (construyó) esta vivienda?
____

H7a. Cuántas habitaciones tiene en total sin contar baño y cocina
__________ Personas

H7a. Cuántas habitaciones tiene en total sin contar baño y cocina
__________ Familias

H13. ¿Tiene energía eléctrica?
1. Sí ☐
2. No ☐

H14. ¿Qué tipo de combustible utiliza para cocinar?
1. Garrafa ☐
2. Gas domiciliario ☐
3. Otro (especifique) ☐

H15. ¿Cómo accede al servicio de internet?
1. Internet fijo ☐
2. Internet móvil ☐
3. Red wifi compartida ☐
4. Otro (especifique) ________________ ☐

H16. ¿Tiene una habitación destinada para cocinar?
1. Sí ☐ → **H16a.**
2. No ☐ → **H17**

H16a. Si la respuesta es sí, la cocina es:
1. Independiente ☐
2. Compartida ☐

H16b. la cocina esta:
1. Dentro de la casa ☐
2. Fuera de la casa ☐

H17. Equipamiento de la Vivienda (aparatos en servicio)

1. Habitación destinada a cocinar con lavaplatos	1. Sí ☐	2. No ☐
2. Habitación destinada a cocinar sin lavaplatos	1. Sí ☐	2. No ☐
3. Refrigerador	1. Sí ☐	2. No ☐
4. Microondas	1. Sí ☐	2. No ☐
5. Teléfono fijo	1. Sí ☐	2. No ☐
6. Teléfono Celular	1. Sí ☐	2. No ☐
7. Televisor	1. Sí ☐	2. No ☐
8. Computadora de escritorio	1. Sí ☐	2. No ☐
9. Computadora portátil	1. Sí ☐	2. No ☐

H18. ¿Ha instalado algún tipo de taller o negocio en su casa?
1. Sí ☐ → **H18a.**
2. No ☐ → **H19.**

H18a. Si la respuesta es sí:
1. Taller ☐
2. Tienda ☐
3. Agencia ☐
4. Oficina ☐
5. Otro (especifique) ________________ ☐

H19. ¿Su familia recibe algún tipo de donación en dinero o producto?
1. Sí, de su familia ☐
2. Sí, de instituciones privadas ☐
3. Sí, de instituciones del estado ☐
4. No recibe ☐
5. Otro (especifique) ________________ ☐

H20. ¿De dónde obtiene agua para beber y cocinar? (marca en orden de importancia 1, 2, 3…)

- Por cañería de red pública dentro de la vivienda ________
- Por cañería de red comunal dentro de la vivienda ________
- Por cañería de red dentro del terreno ________
- Por cañería fuera del lote o terreno ________
- Camión cisterna ________
- Rio, vertiente ________
- Pozo con bomba ________
- Pozo sin bomba ________
- Otro (especifique) ______________________ ________

H21. ¿Su vivienda cuenta con un sistema de almacenamiento de agua?

1. Sí ☐
2. No ☐

G22. ¿Dónde almacena agua?

1. Tanque alto ☐ ________litros
2. Depósito subterráneo ☐ ________litros
3. Mixto ☐ ________litros
4.Otro sistema (especificar)

H23. El agua de red pública o privada se destina a (puede marcar más de una opción):

1. Consumo alimenticio
2. Aseo personal
3. Lavado de ropa
4. Lavado de vehículo automotor
5. jardín o huerto domestico
6. Otros usos domésticos (especifique) ______________________

Tipo de fuente (H20)	Uso
1.	
1.	
1.	

H24. ¿Tiene una conexión domiciliaria de agua POTABLE?

1. Sí ☐
2. No ☐ → **H25**

H25. ¿Por qué no tiene una conexión pública de agua POTABLE? (puede marcar más de una opción)

1. No es posible obtener una conexión ☐
2. No hay red pública de agua cerca de su casa ☐
3. La red abastece cantidades insuficientes ☐
4. El costo de conexión es muy elevado ☐
5. Soy inquilino y el propietario no quiere ☐

H26. ¿Qué problemas cree que le ocasiona el no tener agua potable? (puede marcar más de una opción)

1. Problemas de salud ☐
2. Problemas nutricionales ☐
3. Elevados costos para acceder al agua ☐
4. Contaminación ambiental ☐

H27. ¿Qué piensa usted del abastecimiento de agua por conexión de red?

(1=muy bueno; 5=muy malo).

Calidad	1	2	3	4	5
Cantidad	1	2	3	4	5
Presión	1	2	3	4	5
Costo	1	2	3	4	5
Frecuencia	1	2	3	4	5

H28. Si usa agua del camión cisterna

1. Número de turriles: ________________
2. Costo por turril: ________________
3. Consumo semanal: ________________

H29. ¿Usted cree que la disponibilidad de agua cambió en la última década?

1. Sí, ha disminuido ☐
2. Sí, ha aumentado ☐
3. Se ha mantenido igual (sin cambios) ☐

H30. ¿Ha observado movimientos inusuales en la tierra?

1. Sí ¿Cuándo? (Mes/año)

   Hundimiento ____________________

   Deslizamiento ____________________

   Otro (especifique) ____________________

2. No

H31. ¿Tiene baño o letrina?

1. Sí ☐
2. No ☐

H32. El baño es:

1. Usado por solo un hogar ☐
2. 2. Compartido por otros hogares ☐

H33. El baño tiene desagüe:

1. Red de alcantarillado publica ☐
2. Red de alcantarillado vecinal ☐
3. Cámara séptica ☐
4. Pozo ciego ☐
5. A la calle, río ☐
6. Otro (especifique) ☐

H34. ¿La vivienda tiene un servicio de recolección de basura?

1. Sí ☐
2. No ☐

H35 ¿Dónde bota su basura? (puede marcar más de una opción)

1. Carro basurero ☐
2. Campo libre ☐
3. Lo quema ☐
4. Lo entierra ☐
5. Contenedor ☐
6. Otro (especifique)

   ____________________ ☐

H36. ¿Se organizan entre los vecinos para mantener limpio el barrio?

1. Sí ☐
2. No ☐

¿Cómo?

____

H37. ¿Cuál es el servicio que requiere su barrio con mayor urgencia?

____

I. COMPORTAMIENTO Y PREFERENCIAS DE CONSUMO (Solo para el principal informante)

I1. ¿Quién es el encargado de preparar la comida?
1. Jefe (a) de Hogar ☐
2. Esposo (a) ☐
3. Conviviente ☐
4. Hijo (a) ☐
5. Otro pariente ☐
6. No pariente ☐
7. Empleada cama adentro ☐

I2. ¿Cuántas comidas hace al día?

____

I3. Durante la semana (L-V) su familia suele comer:

	En casa	Fuera de casa
1. Desayuno	☐	☐
2. Almuerzo	☐	☐
3. Cena	☐	☐

I4. Los fines de semana su familia suele comer:

	En casa	Fuera de casa
1. Desayuno	☐	☐
2. Almuerzo	☐	☐
3. Cena	☐	☐

I5. ¿Qué suele beber durante el día en la semana? (respuesta múltiple)
1. Agua ☐
2. Café/infusiones ☐
3. Cerveza ☐
4. Refrescos hervidos azucarados ☐
5. Refrescos hervidos sin azúcar ☐
6. Zumos naturales ☐
7. Gaseosas azucaradas ☐
8. Gaseosas sin azúcar ☐
9. Otro (especifique)

____________________ ☐

I6. ¿Cuál es la comida principal del día?
1. Desayuno ☐
2. Media mañana ☐
3. Almuerzo ☐
4. Merienda ☐
5. Cena ☐
6. Otro (especifique)

____________________ ☐

I7. ¿Quién realiza las compras de alimentos para el hogar?
1. Jefe (a) de Hogar ☐
2. Esposo (a) ☐
3. conviviente ☐
4. Hijo (a) ☐
5. Otro pariente ☐
6. No pariente ☐
7. Empleada cama adentro ☐

I8. ¿Dónde compra sus alimentos? (marque más de una opción)
1. Tienda de barrio ☐
2. Supermercado ☐
3. Micro mercado ☐
4. Mercado (la cancha) ☐
5. Produce sus alimentos ☐ → **I19**
6. Ferias provinciales ☐
7. Ferias de barrio ☐
8. Ferias ecológicas ☐
9. Otro (especifique)

____________________ ☐

I9. ¿Por qué compra sus alimentos en este lugar?

	Supermercado	Cancha
1. Precio	☐	☐
2. Calidad	☐	☐
3. Cercanía	☐	☐
4. Variedad	☐	☐
5. Comodidad	☐	☐
6. Inocuidad	☐	☐
7. Otro	☐	☐

I10. ¿Qué factores considera al momento de elegir sus alimentos?
1. Valor nutricional ☐
2. Información del bien alimentario ☐
3. Precios ☐
4. Preferencias familiares ☐
5. Otro (especifique)

____________________ ☐

I11. Es importante para usted que la comida y los alimentos que consume en un día típico (1=no importante: 5=muy importante):
1. Sean fáciles de preparar
   1 2 3 4 5
2. No requieran tiempo para prepararlos
   1 2 3 4 5

I12. En las preparaciones que consume diariamente utiliza (1=siempre; 5=nunca)

1. Alimentos naturales	1	2	3	4	5
2. Mínimamente procesados	1	2	3	4	5
Ingredientes culinarios (almidones, aceites, azúcar, etc.)	1	2	3	4	5
3. Productos comestibles procesados	1	2	3	4	5
4. Productos comestibles altamente procesados	1	2	3	4	5

I13. Si compra sus alimentos en el mercado (la cancha) ¿Qué clase de alimentos compra?
1. Lácteos y derivados ☐
2. Carnes, huevos y/o pescados ☐
3. Tubérculos ☐
4. Verduras y/o hortalizas ☐
5. Frutas ☐
6. Legumbres ☐
7. Granos ☐
8. Pastas (fideos) ☐
9. Harinas, almidones y/o sémolas ☐
10. Pan y/o derivados ☐
11. Azúcar ☐
12. Aceite, margarina y/u otras grasas ☐
13. Semillas, frutos secos/deshidratada ☐
14. Snacks y bebidas carbonatadas ☐

I14. Si compra sus alimentos en el supermercado ¿Qué clase de alimentos compra?
1. Lácteos y derivados ☐
2. Carnes, huevos y/o pescados ☐
3. Tubérculos ☐
4. Verduras y/o hortalizas ☐
5. Frutas ☐
6. Legumbres ☐
7. Granos ☐
8. Pastas (fideos) ☐
9. Harinas, almidones y/o sémolas ☐
10. Pan y/o derivados ☐
11. Azúcar ☐
12. Aceite, margarina y/u otras grasas ☐
13. Semillas, frutos secos-fruta deshidratada ☐
14. Snacks y bebidas carbonatadas ☐

I15. ¿Quién decide las prioridades de compra de bienes alimentarios del hogar?
1. Esposo ☐
2. Esposa ☐
3. Hijos ☐
4. Hermanos ☐
5. Suegros ☐
6. Toda la familia ☐
7. Otro (especifique)

____________________ ☐

I16. ¿Quién decide las prioridades del gasto familiar
1. Esposo ☐
2. Esposa ☐
3. Hijos ☐
4. Hermanos ☐
5. Suegros ☐
6. Toda la familia ☐
7. Otro (especifique)

____________________ ☐

I17. ¿Cuál es su gasto promedio en alimentos dentro del hogar?

Bs.- ____________________

I18. ¿Cuál es su gasto promedio en alimentos fuera del hogar?

Bs.- ____________________

I19. Si usted cultiva sus alimentos, ¿dónde los cultiva?

1. En su casa ☐
2. En una propiedad zona urbana ☐
3. En una propiedad zona rural ☐
4. Otro (especifique) ____________________ ☐

I20. ¿Qué productos cultiva?

N°	Cultivos en orden de importancia				Cantidad cosecha (carga, caja o Kg)	Destino de la producción				Fuente de riego (represa, pozos, otros)			
	Producto	Superficie (m2)	Mes de Siembra	Mes de cosecha		Semilla	Consumo	Mercado	Lugar venta	Nombre	N° riego prep.	N° riego cultivo	Frecuencia de riego (al mes)
1													
2													
3													
4													
5													
6													

I21. ¿Usted fumiga sus plantaciones?

1. Sí ☐ → **H16a.**
2. No ☐ → **H17**

I22. ¿Quién fumiga?

____

I23. ¿Qué productos utiliza para fumigar?

____

I24. ¿Qué precauciones tiene para realizar el fumigado?

1. Lentes
2. Mascarilla
3. Ropa Especial
4. Otros (especifique)
5. Ninguno

I25. ¿Dónde guarda sus productos para fumigar?

1. Deposito
2. Cocina
3. Dormitorio
4. Otros (especifique)

I26. ¿Tuvo alguna consecuencia en su salud o en la salud de las personas que habitan su hogar debido al uso de productos para fumigar?

1. Sí ☐
2. No ☐

Si la respuesta fue Sí, especifique:

____

I27. ¿Tiene animales de granja?

1. Sí ☐
2. No ☐ → **Sección J**

N°	Animal	Cantidad de animales	¿Con que los alimenta diariamente?	Procedencia del alimento	Costo del alimento (Bs)	Cantidad de Agua (al día)	Procedencia Agua	Encargado Crianza	Destino de los animales	Precio en el mercado (Bs.)
1										
2										
3										

J. FRECUENCIA DE CONSUMO

La intención de esta sección es de registrar el consumo de alimentos y bebidas durante el último mes.
Esta sección debe ser completada por quien prepara los alimentos.

Alimento	Sí	Veces/semana	Veces/mes	Alimento	Sí	Veces/semana	Veces/mes
**J.1. Alimentos naturales**							
Papa				Higo			
Camote				Piña			
Oca				Sandia			
Tomate				Manzana			
Locota				Naranja			
Cebolla				Mandarina			
Zanahoria				Limón			
Brócoli				Guayaba			
Lechuga				Papaya			
Remolacha				Granada			
Achojcha				Palta			
Pepino				Uva			
Ajo				Pacay			
Cilantro				Pera			
Perejil				Kiwi			
Plátano				Otro (especifique)			
**J.2. Mínimamente procesados**							
Huevos				Cañahua			
Huevos de codorniz				Maicena			
Leche pasteurizada				Miel			
Cereales molidos				Carne de res			
Cereales secos				Carne de pollo			
Fruta deshidratada				Carne de pescado			
Frutos secos				Carne de cerdo			
Trigo				Carne de llama			
Quinua				Carne de conejo			
Maíz				Carne de pato			
Avena				Otro (especifique)			
Cebada							
Linaza							

**J.3. Ingredientes culinarios**							
Aceite de girasol				Esencia de vainilla			
Aceite de soya				Azúcar de coco			
Aceite de oliva				Otro (especifique)			
Azúcar de caña							
Sal							
Lenteja							
Maní							
Garbanzo							
**J.4. Alimentos procesados**							
Legumbres en conserva				Te			
Frutas en almíbar				Manzanilla			
Pan				Coca			
Queso				Mantequilla			
Mermeladas				Otro (especifique)			
Atún							
Maíz enlatado							
Cerveza							
Jamón							
Café							
**J.5. Alimentos ultra procesados**							
Gaseosas				Harina fortificada			
Jugos de frutas artificiales				Leche condensada			
Helados				Nuggets de pollo			
Chocolates				Pizza precocida			
Golosinas				Tacos			
Dulces				Nachos			
Galletas				Otro (especifique)			
Papas fritas							
Cereales de caja azucarados							
Panecillos dulces empacados							
Yogurt							
Sopas instantáneas							
Carne de res reconstituida							

K. CONSUMO DE LAS ÚLTIMAS 24 HORAS

El objeto de esta encuesta es conocer el consumo diario de alimentos y bebidas. Anote los alimentos y bebidas, incluyendo el agua, consumidos en las últimas 24 horas.
Escriba el tipo de alimento y la cantidad consumida en medidas caseras o en raciones.

K.1 Desayuno

Hora:	Lugar:
Alimentos (tipo)	Alimento (cantidad)

K.2 Media mañana

Hora:	Lugar:
Alimentos (tipo)	Alimento (cantidad)

K.3 Almuerzo

Hora:	Lugar:
Alimentos (tipo)	Alimento (cantidad)

K.4 Merienda

Hora:	Lugar:
Alimentos (tipo)	Alimento (cantidad)

K.5 Cena

Hora:	Lugar:
Alimentos (tipo)	Alimento (cantidad)

K.6 Otros

Hora:	Lugar:
Alimentos (tipo)	Alimento (cantidad)

DATOS PERSONALES

N° persona	A1. Nombre (Miembros residentes habituales del hogar)	A2. Parentesco o relación con el *Jefe del Hogar*[1]<br>1. Jefe(a) de Hogar<br>2. Esposo(a)<br>3. Conviviente<br>4. Hijo(a)<br>5. Otro pariente<br>6. No pariente<br>7. Empleada cama adentro	A3. Sexo<br>1. Hombre<br>2. Mujer	A4. ¿Cuántos años cumplidos tiene? (Menores de 1 año anote "00")
1			1. Hombre ☐<br>2. Mujer ☐	
2			1. Hombre ☐<br>2. Mujer ☐	
3			1. Hombre ☐<br>2. Mujer ☐	
4			1. Hombre ☐<br>2. Mujer ☐	
5			1. Hombre ☐<br>2. Mujer ☐	
6			1. Hombre ☐<br>2. Mujer ☐	
7			1. Hombre ☐<br>2. Mujer ☐	

L. REPRESENTACION GRAFICA DE PRINCIPAL COMIDA HABITUAL.

Por favor dibuje cómo se ve, habitualmente, su(s) plato(s) de alimentos de su comida principal:

OBSERVACIONES

NOTAS

SAN SIMON UNIVERSITY
CENTER OF PLANNING AND MANAGEMENT
NUTRITION AND DEVELOPMENT SURVEY

PRESENTATION

The objective of this survey is to provide up-to-date information about the socio-economic, cultural and biogeographical conditions of the population of the municipality of Cochabamba, related to the state of nutrition and health, and their potential for improvement; así como su vinculación con otras variables que influyen en la calidad de vida de su población.

CONFIDENTIALITY

The responses that we bring will be treated with the greatest confidentiality possible and, later, grouped with the other collaborators with the study. Muchas gracias.

MUNICIPALITY	DISTRICT	ZONE	BLOCK	HOUSE	HOUSEHOLD	NUMBER OF HOUSEHOLDS IN THE HOUSE	OBSERVATIONS

SURVEYOR COD	
COD SUPERVISOR	
COD CODIFIERS	
DIGITIZER COD	
COD TICKET	
DATE	

A. SOCIODEMOGRAPHIC CHARACTERISTICS

No. the person	A1. Name (Usual household residents)	A2. Relationship to the *Head* of Household[1]<br><br>1. Head of Household<br>2. Spouse<br>3. Cohabitant<br>4. Child<br>5. Other relative<br>6. No relative<br>7. Live-in maid	A3. Sex<br><br>1. Man<br>2. Woman	A4. How old is he/she? (For children under 1 year old, write "00")	A5. Can you read and write?	A6. What is your marital status:<br><br>1. Married<br>2. Cohabitant<br>3. Separated<br>4. Divorced<br>5. Widow<br>6. Single	A7. What was the highest level and course of instruction you attained?<br>1. Primary<br>2. Secondary<br>3. Middle technician<br>4. Senior technician<br>5. Military Institute<br>6. Police Institute<br>7. Teacher training<br>8. University<br>9. Postgraduate diploma<br>10.Postgraduate Master's degree<br>11.Postgraduate doctorate	A8. The establishment where he/she studies (or the last one where he/she studied) is (was):	A9. During this year, did you enroll in any courses, grades in school education, alternative education, higher education, or postgraduate studies?	A10. What idioms does he/she speak? (multiple choice)<br><br>1. Spanish<br>2. Aymara<br>3. Quechua<br>4. Guarani<br>5. Other (specify)<br>6. Foreign (English , French, German, Portuguese, etc.)
1			1. Man ☐<br>2.Women ☐		1. Yes ☐<br>2.No ☐		Course:________________<br><br>Level:________________	1. Public ☐<br>2. Private ☐<br>3. Did not attend ☐	1. Yes ☐<br>2. No ☐	
2			1. Man ☐<br>2.Women ☐		1. Yes ☐<br>2.No ☐		Course:________________<br><br>Level:________________	1. Public ☐<br>2. Private ☐<br>3. Did not attend ☐	1. Yes ☐<br>2. No ☐	
3			1. Man ☐<br>2.Women ☐		1. Yes ☐<br>2.No ☐		Course:________________<br><br>Level:________________	1. Public ☐<br>2. Private ☐<br>3. Did not attend ☐	1. Yes ☐<br>2. No ☐	
4			1. Man ☐<br>2.Women ☐		1. Yes ☐<br>2.No ☐		Course:________________<br>Level:________________	1. Public ☐<br>2. Private ☐<br>3. Did not attend ☐	1. Yes ☐<br>2. No ☐	
5			1. Man ☐<br>2.Women ☐		1. Yes ☐<br>2.No ☐		Course:________________<br>Level:________________	1. Public ☐<br>2. Private ☐<br>3. Did not attend ☐	1. Yes ☐<br>2. No ☐	
6			1. Man ☐<br>2.Women ☐		1. Yes ☐<br>2. No ☐		Course:________________<br>Level:________________	1. Public ☐<br>2. Private ☐<br>3. Did not attend ☐	1. Yes ☐<br>2. No ☐	

B. MIGRATION

Number of	B1. Where was he born?<br><br>1. Country<br>2. Province<br>3. Municipality<br>4. Location<br>5. District	B2. Where did you live 5 years ago (2018)?<br>1. Here→ **B4**<br>2. In another place→ **B3**<br>2a. Country<br>2b. Province<br>2c. Municipality<br>2d. Locality<br>2nd District	B3. What was the reason he left that place?<br>1. Job search<br>2. Job transfer<br>3. Education<br>4. Health<br>5. Family reason<br>6. Another reason (specify)	B4. How long (in years) have you lived in or returned to this place?	B5. ¿Where did you live immediately before your current place of residence?<br>1. Country<br>2. Province<br>3. Municipality<br>4. Location<br>5. District	B6. Where do your parents and grandparents live?<br><br>1. Country<br>2. Province<br>3. Municipality<br>4. Location<br>5. District	B7. Why did you or your family choose that place?<br>1. I had relatives or acquaintances<br>2. Educational opportunities<br>3. Job opportunities<br>4. Work before in the place<br>5. Improved living conditions<br>6. I had a secure job<br>7. For family reasons<br>8. Others (specify)	B8. Do you have any relatives abroad?<br><br>1. Yes → **B8**<br>2. No → **Section C**	B9. In which country? Specify the year of migration<br><br>1. Country<br>2. Year of migration	B10. What is their relationship to each other?<br><br>1. Head of Household<br>2. Spouse<br>3. Cohabitant<br>4. Son (a)<br>5. Other relative
1	1……………………<br>2……………………<br>3……………………<br>4……………………<br>5……………………	2a……………………<br>2b……………………<br>2c……………………<br>2d…………………… ……. .<br>2nd…………………… ……. .			1……………………<br>2……………………<br>3……………………<br>4……………………<br>5……………………	1……………………<br>2……………………<br>3……………………<br>4……………………<br>5……………………		1. Yes ☐<br>2. No ☐	1……………………<br>2……………………	
2	1……………………<br>2……………………<br>3……………………<br>4……………………<br>5……………………	2a……………………<br>2b……………………<br>2c……………………<br>2d…………………… ……. .<br>2nd…………………… ……. .			1……………………<br>2……………………<br>3……………………<br>4……………………<br>5……………………	1……………………<br>2……………………<br>3……………………<br>4……………………<br>5……………………		1. Yes ☐<br>2. No ☐	1……………………<br>2……………………	
3	1……………………<br>2……………………<br>3……………………<br>4……………………<br>5……………………	2a……………………<br>2b……………………<br>2c……………………<br>2d…………………… ……. .<br>2nd…………………… ……. .			1……………………<br>2……………………<br>3……………………<br>4……………………<br>5……………………	1……………………<br>2……………………<br>3……………………<br>4……………………<br>5……………………		1. Yes ☐<br>2. No ☐	1……………………<br>2……………………	
4	1……………………<br>2……………………<br>3……………………<br>4……………………<br>5……………………	2a……………………<br>2b……………………<br>2c……………………<br>2d…………………… ……. .<br>2nd…………………… ……. .			1……………………<br>2……………………<br>3……………………<br>4……………………<br>5……………………	1……………………<br>2……………………<br>3……………………<br>4……………………<br>5……………………		1. Yes ☐<br>2. No ☐	1……………………<br>2……………………	
5	1……………………<br>2……………………<br>3……………………<br>4……………………<br>5……………………	2a……………………<br>2b……………………<br>2c……………………<br>2d…………………… ……. .<br>2nd…………………… ……. .			1……………………<br>2……………………<br>3……………………<br>4……………………<br>5……………………	1……………………<br>2……………………<br>3……………………<br>4……………………<br>5……………………		1. Yes ☐<br>2. No ☐	1……………………<br>2……………………	
6	1……………………<br>2……………………<br>3……………………<br>4……………………<br>5……………………	2a……………………<br>2b……………………<br>2c……………………<br>2d…………………… ……. .<br>2nd…………………… ……. .			1……………………<br>2……………………<br>3……………………<br>4……………………<br>5……………………	1……………………<br>2……………………<br>3……………………<br>4……………………<br>5……………………		1. Yes ☐<br>2. No ☐	1……………………<br>2……………………	

C. EMPLOYMENT (FOR ALL PEOPLE AGED 5 AND OVER)				ONLY FOR WORKERS			ONLY FOR THE UNEMPLOYED AND JOB SEEKERS		
**No. person**	**C1. What was your main labor activity during the past week?**<br><br>1. Worked<br>2. He didn't work, but he had vacation<br>3. He looked for work (unemployed) **C4**<br>4. Looking for work for the first time<br>5. Housework and worked<br><br>6. Only work from house<br>7. He/She only studied<br>8. Is retired or rent list **C2**<br>9. Other (specify)	**C2. Did you engage in any activity that provided income for your family and yourself?**<br><br>1. Yes<br>2. No	**C3. Did you do anything to find work?**<br>1. Consultation with employers.<br>2. Attended a job interview<br>3. Answered to job advertisement<br>4. I consulted on the internet<br>5. I consult with friends, relatives, or acquaintances<br>6. I consult newspapers<br>7. I posted my resume on social media<br>8. I did nothing→ **D1**	**C4. What was your main occupation last week? (or his last occupation if he was unoccupied)**<br><br>26301 Economists<br>42206 Receptionist<br>51200 Cooks<br>95102 Lustre repair<br>91101 Domestic workers<br><br>***Use COB codes***	**C5. In your main occupation you work as:**<br><br>1. Employee<br>2. Laborer<br>3. Worker on your own account<br>4. TCP subordinate to a single company or capital<br>5. Unpaid family member<br>6. Patron o employer<br>7. Independent professional<br>8. Domestic employee	**C6. What did the establishment you work for (or last worked for) do?**<br><br>47921 Street vendor<br>49222 Transportation by taxi, radio taxi and motorcycle taxi<br>***Use the CAEB codes***	**C7. Why did he leave his previous occupation?**<br><br>1. Contract termination<br>2. Staff cuts<br>3. Found a better job<br>4. Other (specify)	**C8. In your previous occupation you worked as:**<br><br>1. Employee<br>2. Laborer<br>3. Worker on your own account<br>4. TCP subordinate to a single company or capital<br>5. Unpaid family member<br>6. Patron o employer<br>7. Independent professional<br>8. Domestic employee	**C9. In what field would you like to perform duties?**<br><br>47921 Street vendor<br>49222 Transportation by taxi, radio taxi and motorcycle taxi<br>***Use the CAEB codes***
1									
2									
3									
4									
5									
6									

C. EMPLOYMENT (ONLY EMPLOYED AND UNEMPLOYED)										
N ° person	C10. His work is (was):<br><br>1. Permanent<br>2. Temporal<br>3. Occasional	C11. Do you have a contract of employment?<br><br>1. Yes, for 3 months.<br>2. Yes , for a fixed period<br>3. Yes, of indefinite duration.<br>4. He/She does not have (had) a contract.	C12. What do you receive (did you receive) at the establishment where you work (did you work)? (you can mark more than one option):<br><br>1. Christmas bonus<br>2. Vacations<br>3. Social security care<br>4. Compensation<br>5. None of the above	C13. The income he/she earned from his/her work is:<br><br>1. Fixed salary<br>2. Fixed salary and percentage<br>3. Piecework or percentage<br>4. Daily<br>5. Income from self-employment<br>6. He does not receive monetary compensation<br>7. Others (specify)	C14. Normally, how many days and hours did you work (or did you work) per week?		C15. How much do I normally earn?<br><br>(Ask about net or disposable income)		C16. Approximately how many people worked at the establishment during the last week? (Including yourself)
					Days	Hours/day	Frequency<br>1. Per day<br>2. Per week<br>3. Per month<br>4. Per year	Amount (Bs.-)	
1									
2									
3									
4									
5									
6									

D. FAMILY HISTORY AND HEALTH

No. person	D1. Does your family have access to a health insurance system?<br>1. National public insurance<br>2. Private insurance<br>3. Other (specify)<br>4. Doesnt have	D2. Mainly, where do you go in case of illness?<br>1. Hospital<br>2. Private Clinic<br>3. Insurance<br>4. Private consulting room<br>5. Health center<br>6. Yatiri<br>7. Jampiq<br>8. Healer<br>9. Buy at the pharmacy without a prescription<br>10. Home medicine<br>11. Other (specify)	D3. What are the most frequent health problems in your family?<br>1. Cold<br>2. Cough<br>3. Diarrhea<br>4. Infections<br>5. Others (specify)	D4. Has your doctor diagnosed you with any of the following diseases?<br>1. Ischemic disease<br>2. Diabetes mellitus<br>3. Diabetes insipidus<br>4. Hypertension<br>5. Osteoporosis<br>6. Obese<br>7. Cancer<br>8. Fatty liver<br>9. Chronic kidney disease<br>10. Thyroid problem<br>11. Malabsorption syndrome<br>12. Respiratory diseases<br>13. Autoimmune disease Wich one?<br>14. No disease<br>15. Other (specify)	D5. Do your parents suffer or have they suffered from any of the following illnesses?<br>1. Ischemic disease<br>2. Diabetes mellitus<br>3. Diabetes insipidus<br>4. Hypertension<br>5. Osteoporosis<br>6. Obese<br>7. Cancer<br>8. Fatty liver<br>9. Chronic kidney disease<br>10. Thyroid problem<br>11. Malabsorption syndrome<br>12. Respiratory diseases<br>13. Autoimmune disease Wich one?<br>14. No disease<br>15. Other (specify)	D6. Do you follow any special diet?<br>1. Yes<br>2. No→ **E1**<br>3. Don't know→ **E1**	D6a. What does their diet consist of?<br>**(multiple response)**<br>1. Vegetarian<br>2. Vegan<br>3. Low in calories<br>4. Low in fat<br>5. Low in carbohydrates<br>6. Low in animal proteins<br>7. High in fat or keto<br>8. High in protein<br>9. Gluten-free<br>10. Liquid or mixed<br>11. Low in sodium<br>12. Omit processed food<br>13. Skip sodas and sugar<br>14. Omit dairy products<br>15. Omit flour .<br>16. Omit trans fats<br>17. Other (specify)	D7. How long have you been on this diet?<br>**(Weeks)**	D8. Who prescribed or recommended the diet?<br>1. Doctor<br>2. Family<br>3. Friend<br>4. Neighbor<br>5. Healer<br>6. Internet<br>7. Nobody<br>8. Other (specify)
1									
2									
3									
4									
5									
6									

E. FERTILITY AND INFANT MORTALITY: ONLY FOR WOMEN OF REPRODUCTIVE AGE 12 YEARS AND OVER

Person number	E1. How many live births have you had?<br><br>__________ sons and daughters	E2. Of these, how many are alive?	E3. In what month and year was your last son or daughter born alive?		E4. Is this last child alive?<br><br>1. Lives→ **E6**<br>2. Death→ **E5**	E5. On what date did he die?		E6. Who attended your last delivery?<br>1. Doctor<br>2. Nurse<br>3. Midwife<br>4. Family<br>5. Other (specify)
			**Month**	**Year**		**Month**	**Year**	
1								
2								
3								
4								
5								
6								

F . CONSUMER BEHAVIOR AND PREFERENCES OUTSIDE THE HOME (Household Members)									
**No. the person**	**F1. When you eat out, what do you usually choose most often?:**<br>1. Homemade food<br>2. Salads<br>3. Traditional food<br>4. Hamburgers<br>5. Grilled meats<br>6. Deep-fried<br>7. Empanadas<br>8. Cakes, pastries, ice cream<br>9. Other (specify)	**F1a. Where?**<br>1. Street vendor<br>2.Market stall<br>3. Restaurant<br>4. University cafeteria<br>5. Kiosk<br>6. A friend's house<br>7. Other (specify)	**F1b. With whom?**<br>1.With friends<br>2.With my partner<br>3.With my co-workers<br>4.Eats alone<br>5.Other (specify)	**F2. Indicate the types of food you consume most (ordinal).**<br>1. Natural foods.<br>2. Minimally processed.<br>3. Culinary ingredients.<br>4. Processed foods.<br>5. Ultra-processed foods.	**F3. Of the following options, which attributes do you consider most important in your food?**<br>**Determine an order of importance.**<br>a) Be Nutritious<br>b) Be rich in protein<br>c) They are rich in fiber	**F4. Of the following options, which attributes do you consider most important in your food?**<br>**Determine an order of importance.**<br>a) They are low in calories<br>b) They are low in sugar<br>c) They are low in fat.	**F5. Of the following options, which attributes do you consider most important in your food?**<br>**Determine an order of importance.**<br>a) They are from a well-known place or brand<br>b) Have good taste<br>c) Have a good presentation<br>d) Have a low price<br>e) They help me control my weight<br>f) Contain natural ingredients	**F6. Of the following options, which is your favorite?**<br>1. Hamburger<br>2. Pizza<br>3. Fried chicken<br>4. Salchipapa<br>5. Grilled meats<br>6. Other (specify)	**F7. What foods do you prefer not to consume?**
1				1)________<br>2)________<br>3)________	1)________<br>2)________	1)________<br>2)________	1)________<br>2)________		
2				1)________<br>2)________<br>3)________	1)________<br>2)________	1)________<br>2)________	1)________<br>2)________		
3				1)________<br>2)________<br>3)________	1)________<br>2)________	1)________<br>2)________	1)________<br>2)________		
4				1)________<br>2)________<br>3)________	1)________<br>2)________	1)________<br>2)________	1)________<br>2)________		
5				1)________<br>2)________<br>3)________	1)________<br>2)________	1)________<br>2)________	1)________<br>2)________		
6				1)________<br>2)________<br>3)________	1)________<br>2)________	1)________<br>2)________	1)________<br>2)________		
7				1)________<br>2)________<br>3)________	1)________<br>2)________	1)________<br>2)________	1)________<br>2)________		

UNIT OF ANALYSIS: HOME

G. MORTALITY

G1. From January 2019 to date, has anyone who lived with you in this household died?
1. Yes □ **How many people?** _______________→ **G2.**
2. No □ → **Section H.**

Person number	G2. Name of the person.	G3. Date of death		G4. How old were they when they died?	G5. The person was: 1. Man 2. Woman	G6. Do you have a death certificate? 1. Yes 2. No	G7. Did he die from COVID-19? 1. Yes 2. No	G8. Died for some cause related to: (you can mark more than one option) 1. Ischemic heart disease 2. Diabetes 3. Hypertension 4. Osteoporosis 5. Obesity 6. Malnutrition 7. Cancer 8. Chronic liver disease 9. Chronic kidney disease 10. Thyroid problem 11. Malabsorption syndrome 12. Respiratory diseases 13. Suicide 14. Natural cause 15. Other (specify)
		Month	Year					
1								
2								
3								
4								

H. HOUSING (For the main informant only)				
**H1. Type of housing:**<br>1. Independent house<br>2. Department<br>3. Loose room(s) in a tenement house<br>4. Hut<br>5. Makeshift housing<br>6. Premises intended for residential use<br>7. Other (specify) _____________	**H2. Walls**<br>1. Adobe with coating<br>2. Adobe without coating<br>3. Brick<br>4. Stone<br>5. Block the cement<br>6. Cane, palm tree, others<br>7. Other (specify) _____________	**H3. Floor**<br>1. Wood<br>2. Mosaic or tiles<br>3. Brick<br>4. Cement<br>5. Soil<br>6. Other (specify)<br>_____________	**H4. Ceiling**<br>1. Ceramic tile<br>2. Fiber cement tile<br>3. Concrete<br>4. Calamine<br>5. Clay tile<br>6. Straw, cane or palm<br>7. Other (specify)<br>_____________	**H5. Inner Ceiling**<br>1. Plaster blocks<br>2. Plastered<br>3. Lying down (cloth)<br>4. Does not have<br>5. Other (specify)<br>_____________
**H6. This house is:**<br>1.Own already paid→ **H9**<br>2.Owns it and is paying for it→ **H9**<br>3.Rented→ **H8**<br>4.Anticretic→H **8**<br>5.Provided or transferred for service<br>6.Other form of possession (please specify)<br>___________	**H7. Indicate the number of rooms JUST to sleep:**<br>__________ Rooms<br>**H7a. How many rooms does it have in total, not including the bathroom and kitchen?**<br>__________ Rooms	**H8. How much do you pay monthly in rent or mortgage payments?**<br>**(For anticretic contracts, please also register the fixed amount of the contract.)**<br>Bs.- ___________ Rented Amortization<br>Bs.- ____________ Fixed mount Anticretic	**H9. How did he get this house?**<br>1. I buy it ready-made<br>2. He ordered it to be built<br>3. He built it little by little<br>4. Inheritance or donation<br>5. Credit<br>6. Remittances<br>7. Other (Specify)<br>_____________	**H10. In what year did you acquire (build) this house?**<br>__________________<br>**H11. How many people live in your home?**<br>__________ People<br>**H12. How many families live in your home?**<br>__________ Families
**H13. Do you have electric energy?**<br>1.Yes 2. No<br>**H14. What type of fuel do you use for cooking?**<br>1. Gas canister<br>2. Domestic gas<br>3. Other (spesify) ____________<br>**H15. How do I access the internet service?**<br>1. LAN connection<br>2. Mobil Internet<br>3. Shared wifi network<br>4. Other (spesify) ____________	**H16. Do you have a room designated for cooking?**<br>1. Yes → **H16a.**<br>2. No → **H17**<br>**H16a. If the answer is yes, the kitchen is:**<br>1. Independent<br>2. Shared<br>**H16b. the kitchen is:**<br>1. Inside the house<br>2. Outside the house	**H17. Household Equipment (appliances in service)**<br>1. Kitchen with sink<br>2. Kitchen without sink<br>3. Refrigerator<br>4. Microwave<br>5. Landline phone<br>6. Cell phone<br>7. Television<br>8. Desktop computer<br>9. Laptop computer	**H18. ¿Ha installed algún tipo de taller o negocio en su casa?**<br>1. Yes □→ **H18a.**<br>2. No □→ **H19.**<br>**H18a. If the answer is yes :**<br>1. Workshop<br>2. Store<br>3. Agency<br>4. Office<br>5. Other (please specify)<br>_____________	**H19. Does your family receive any type of donation in money or product?**<br>1. Yes, from family<br>2. Yes, from private institutions<br>3. Yes, from state institutions<br>4. Does not receive<br>5. Other (please specify)_____________

H 20. Where do you get your drinking and cooking water from? (Mark in order of importance 1, 2, 3…)

- Through public water main inside the dwelling_______
- Through communal water main inside the dwelling______
- Through mains water within the property _______________
- Through pipe outside the lot or property _________________
- Water truck ________________________________
- River, spring __________________________________
- Well with pump________________________________
- Well without pump______________________________
- Other (specify)_____________________

H21. Does your home have a water storage system?

1.Yes □
2.No □

G22. Where do you store water?

1. High-level tank □ __________ liters
2. Underground tank □ __________ liters
3. Mixed system □ __________ liters
4. Other system (specify) □ __________ liters

H23. Water from public or private mains is used for (you can select more than one option):

1. Food consumption
2. Personal hygiene
3. Laundry
4. Vehicle washing
5. Home garden or orchard
6. Other domestic uses
(specify) __________________

Source type (H20)	Use
1.	
2.	
3.	

H24. Do you have a home drinking water connection?

1. Yes □
2. No □ →H25

H25. Why doesn't it have a public drinking water connection? (you can mark more than one option)

1. It is not possible to obtain a connection
2. There is no public water network near your house
3. The network supplies insufficient quantities
4. The connection cost is too high
5. I am a tenant and the landlord doesn't want to connect me

H26. What problems do you think the lack of drinking water causes you? (You can mark more than one option.)

1. Health problems □
2. Nutritional problems □
3. High costs of accessing water □
4. Environmental pollution □

H27. What do you think of the mains water supply?

(1=very good; 5=very bad).

Quality 1 2 3 4 5
Quantity 1 2 3 4 5
Pressure 1 2 3 4 5
Cost 1 2 3 4 5
Frequency 1 2 3 4 5

H28. If you use water from a tanker truck

1. Number of barrels: ___________
2. Cost per barrel: ______________
3. Weekly consumption: ___________

H29. Do you think the availability of water changed in the last decade?

1. Yes, it has decreased □
2. Yes, it has increased □
3. It has remained the same (no changes)□

H30. Have you observed any unusual movements on Earth?

1. Yes **When ? (Month/year)**
Subsidence ________________________
Sliding ________________________
Other (specify) ________________________

2. No

H31. Do you have a bathroom or toilet?

1. Yes □
2. No □

H32. The bathroom is:

1. Used by only one household □
2. Shared by other households□

H33. The bathroom has a drain:

1. Public sewer system □
2. Neighborhood sewer system □
3. Septic tank □
4. Cesspool □
5. To street, river □
6. Other (specify)

H34. Does the property have a garbage collection service?

1. Yes □
2. No □

H35 Where do you throw your trash? (you can mark more than one option)

1. Garbage truck □
2. Open field □
3. Burns it □
4. Buries it □
5. Container □
6. Other (specify) □

H36. Do the neighbors organize themselves to keep the neighborhood clean?

1. Yes □
2. No □

How?
____

H37. What service does your neighborhood need most?

____

I . CONSUMER BEHAVIOR AND PREFERENCES (For the main informant only)

I1. Who is in charge of preparing the food?

1. Head of Household
2. Spouse
3. Partner
4. Child
5. Other Relative
6. Non-Relative
7. Live-in Domestic Worker

I2. How many meals does he/she eat per day?

____

I3. During the week (M-F) your family usually eats:

	At house	Outside from home
1.Breakfast	□	□
2.Lunch	□	□
3.Dinner	□	□

I4. On weekends, his family usually eats:

	At house	Outside from home
1.Breakfast	□	□
2.Lunch	□	□
3.Dinner	□	□

I5. What do you usually drink during the day on a weekday? (multiple choice)

1. Water □
2. Coffee/Tea □
3. Beer □
4. Sweetened Boiled Drinks □
5. Unsweetened Boiled Drinks □
6. Natural Juices □
7. Sugary Sodas □
8. Unsweetened Sodas □
9. Other (please specify) □

I6. What is the main meal of the day?

1. Breakfast
2. Half morning
3. Lunch
4. Snack
5. Dinner
6. Other (specify)

I7. Who does the grocery shopping for the household?

1. Head of Household
2. Spouse
3. Partner
4. Child
5. Other Relative
6. Non-Relative
7. Live-in Domestic Worker

I8. Where do you buy your food?

(Please select more than one option)

1. Neighborhood store □
2. Supermarket □
3. Mini-market □
4. Market □
5. Produces their own food□→ **I19**
6. Provincial fairs □
7. Neighborhood fairs □
8. Organic fairs □
9. Other (specify) □ ___________

I9. Why do you buy your food here?

	Supermarket	Market
1. Price	□	□
2. Quality	□	□
3. Neighborhood	□	□
4. Variety	□	□
5. Comfort	□	□
6. Safety	□	□
7. Other	□	□

I10. What factors do you consider when choosing your food?

1. Nutritional value □
2. Food product information □
3. Prices □
4. Family preferences □
5. Other (please specify) □

____

I11. It is important to you that the food and ingredients you consume on a typical day (1=not important: 5=very important):

1. They should be easy to prepare 1 2 3 4 5
2. They don't require time to prepare. 1 2 3 4 5

I12. In the preparations he consumes daily he uses (1=always; 5=never)

1. Natural foods 1 2 3 4 5
2. Minimally processed 1 2 3 4 5
   Culinary ingredients 1 2 3 4 5
   (starches, oils, sugar, etc.).
3. Processed edible products 1 2 3 4 5
4. Highly processed edible products 1 2 3 4 5

I13. If you buy your food at the market (the court), what kind of food do you buy?

1. Dairy products
2. Meat, eggs, and/or fish
3. Tubers
4. Vegetables
5. Fruits
6. Legumes
7. Grains
8. Pasta (noodles)
9. Flours, starches, and/or semolina
10. Bread and/or bread products
11. Sugar
12. Oil, margarine, and/or other fats
13. Seeds, nuts/dried fruit
14. Snacks and carbonated beverages

I14. If you buy your food at the supermarket, what kind of food do you buy?

1. Dairy products
2. Meat, eggs, and/or fish
3. Tubers
4. Vegetables
5. Fruits
6. Legumes
7. Grains
8. Pasta (noodles)
9. Flours, starches, and/or semolina
10. Bread and/or bread products
11. Sugar
12. Oil, margarine, and/or other fats
13. Seeds, nuts/dried fruit
14. Snacks and carbonated beverages

I15. Who decides the household's food purchasing priorities?

1. Husband □
2. Spouse □
3. Children □
4. Brothers □
5. Parents-in-law □
6. The whole family □
7. Other (specify) □

I16. Who decides family spending priorities?

1. Husband □
2. Spouse □
3. Children □
4. Brothers □
5. Parents-in-laws □
6. The whole family □
7. Other (specify) □

I17. What is your average household food expenditure?

Bs.- ______________________________

I18. What is your average expenditure on food outside the home?

Bs.- ______________________________

J. FREQUENCY OF CONSUMPTION

The intention of this section is to register the consumption of food and beverages during the last month.
This section must be completed by the person who prepares the food.

Food	Yes	Times/week	Times/month	Food	Yes	Times/week	Times/month
**J.1. Natural foods**							
Potato				Fig			
Sweet potato				Pineapple			
Oca				Watermelon			
Tomato				Apple			
Bell pepper				Orange			
Onion				Tangerine			
Carrot				Lemon			
Broccoli				Guava			
Lettuce				Papaya			
Beetroot				Pomegranate			
Achojcha (a type of squash)				Avocado			
Cucumber				Grape			
Garlic				Pacay			
Cilantro				Pear			
Parsley				Kiwi			
Plantain				Other (please specify)			
**J.2. Minimally processed**							
Eggs				Cañahua			
Quail eggs				Cornstarch			
Pasteurized milk				Honey			
Ground cereals				Beef			
Dried cereals				Chicken			
Dried fruit				Fish			
Nuts				Pork			
Wheat				Llama			
Quinoa				Rabbit			
Corn				Duck			
Oats				Other (please specify)			

Barley							
Flaxseed							
**J.3. Culinary ingredients**							
Sunflower oil				Vanilla essence			
Soybean oil				Coconut sugar			
Olive oil				Other (specify)			
Cane sugar							
Salt							
Lentil							
Peanut							
Chickpeas							
**J.4. Processed foods**							
Canned legumes				Tea			
Fruits in syrup				Chamomile			
Bread				Coca			
Cheese				Butter			
Jams				Other (specify)			
Tuna							
Canned corn							
Beer							
Ham							
Coffee							
**J.5. Ultra-processed foods**							
Soft drinks				Fortified flour			
Artificial fruit juices				Condensed milk			
Ice cream				Chicken nuggets			
Chocolates				Pre-cooked pizza			
Candy				Tacos			
Sweets				Nachos			
Cookies				Other (please specify)			
Potato chips							
Sugary boxed cereals							

Packaged sweet rolls							
Yogurt							
Instant soups							
Reconstituted beef							

The object of this survey is to know the daily consumption of food and drinks. Write down the food and beverages (including water) consumed in the last 24 hours. Write down the type of food and the amount consumed in household measures or in portions.

K.1 Breakfast

Time:	Place:
**Food (type)**	**Food (quantity)**

K.2 Mid-morning

Time:	Place:
**Food (type)**	**Food (quantity)**

K.3 Lunch

Time:	Place:
**Food (type)**	**Food (quantity)**

K.4 Snack

Time:	Place:
**Food (type)**	**Food (quantity)**

K.5 Dinner

Time:	Place:
**Food (type)**	**Food (quantity)**

K.6 Others	
**Time:**	**Place:**
**Food (type)**	**Food (quantity)**

L. GRAPHIC REPRESENTATION OF MAIN USUAL MEAL.

Please draw what your main meal plate(s) usually look like.:
